# Dawn of the dread: threatening cinematic virtual reality environments enhance general but not specific pavlovian-instrumental transfer

**DOI:** 10.3389/fnbeh.2026.1803377

**Published:** 2026-06-16

**Authors:** Shaira Berg, Felippe Espinelli Amorim, Emily Breese, Anna Yew, Adam Shea, Thea Hardman, Zofia Zatyczyc, Srinidhi Krishnan, Trevor W. Robbins, Sharon Morein-Zamir, Amy L. Milton

**Affiliations:** 1 Department of Psychology, University of Cambridge, Cambridge, United Kingdom; 2 Department of Psychiatry and Behavioral Sciences, University of Texas at Austin, Austin, TX, United States; 3 School of Psychology and Sports Science, Anglia Ruskin University, Cambridge, United Kingdom

**Keywords:** appetitive, aversive, immersion, learning, Pavlovian instrumental transfer, reinforcement, threat, virtual reality

## Abstract

**Introduction:**

Pavlovian-Instrumental Transfer (PIT) exemplifies how Pavlovian-motivational influences modulate goal-directed behavior, yielding outcome-specific (specific PIT) and general (general PIT) transfer. General PIT is commonly interpreted as outcome-general invigoration and is sensitive to stress. However, human PIT research typically uses visual, appetitive procedures, whereas rodent PIT research often uses auditory cues, limiting translation. We tested whether cue modality, along with additional factors such as immersive threat contexts, modulates PIT, and whether virtual reality (VR) enhances general transfer.

**Methods:**

Across three experiments (*N* = 196), participants completed a PIT task: (1) two-dimensional (2D) appetitive PIT with auditory vs. visual cues (Experiment 1; *n* = 60); (2) VR PIT comparing appetitive (positive reinforcement) vs. aversive (negative reinforcement; “zombie”) contexts (Experiment 2; *n* = 40); and (3) aversive VR PIT preceded by immersive compound threat scenarios (neutral, spiders, contamination) in individuals stratified by contamination fear (CF) (Experiment 3; *n* = 96). Specific and general PIT were computed from baseline-corrected response rates. Stress induction (Experiment 3) was assessed using photoplethysmography-derived heart rate variability (HRV), salivary alpha-amylase (sAA), and self-report measures.

**Results:**

Robust specific (*η*_p_^2^ > 0.45) and general PIT (*η*_p_^2^ > 0.68) were observed across all experiments. In Experiment 1, PIT magnitude did not differ by cue modality; specific PIT exceeded general PIT across auditory and visual conditions. In Experiment 3, threat scenario type and contamination fear did not significantly alter transfer effects. Nevertheless, increased stress indices were observed, including phase-dependent HRV changes, elevated sAA from pre- to post-test, and higher self-reported anxiety (with stronger subjective fear/disgust in the contamination condition).

**Discussion:**

Across experiments, general PIT was larger in the VR studies than in the 2D study, whereas specific PIT remained stable; however, VR was not manipulated independently of reinforcement context and other procedural differences, precluding strong causal inference about its effect on general PIT. Despite this, human PIT appeared robust across cue modality and reinforcement context, and there was an indication that VR immersion may selectively amplify general invigoration while sparing specific PIT across all three experiments, albeit with a small effect (pseudo-*R*^2^ = 0.06).

## 1 Introduction

Pavlovian-Instrumental Transfer (PIT) refers to previously learned Pavlovian stimuli influencing instrumental action and allows insight into the motivational underpinnings of reward-oriented behavior (Cartoni et al., 2016). PIT can be observed when the instrumental action is learned either before or after the Pavlovian stimuli, and it occurs when a previously learned association between a conditioned stimulus (CS) and an unconditioned stimulus (US) interacts with responding for a US. This increases motivation to respond for the same or similar rewards (Dinsmoor, 1983). PIT occurs in both specific and general forms. Specific PIT is observed when a Pavlovian CS and instrumental response share a common US, such that the presentation of the US-associated CS enhances subsequent instrumental responding. General PIT is observed when the presentation of a CS associated with a US of similar valence enhances instrumental responding (Degni et al., 2022). General PIT has been linked to outcome-general behavior that can underpin maladaptive habit formation, as it elicits cue-oriented responding regardless of reward outcome, even when the US is devalued (Holland, 2014; Robbins et al., 2012). This is suggested to occur because automatic and intrinsic learning processes are recruited in this type of responding (Belanger et al., 2022). By contrast, specific PIT has been linked to goal-directed behavior, as it elicits cue-oriented responding for a specific outcome (Hinojosa-Aguayo and González, 2020). PIT can be measured using both positive reinforcers, that is, responding for rewards (Corbit and Balleine, 2011; Watson et al., 2014; Kirsten et al., 2021; Verhoeven et al., 2018; Cartoni et al., 2015) and negative reinforcers (Campese et al., 2013). Negative reinforcement refers to behavior being reinforced or strengthened by the avoidance of an aversive outcome and is hypothesized to play a role in the development and maintenance of conditions such as obsessive-compulsive disorder (OCD; Gerlicher and Kindt, 2020). Moreover, PIT offers a method of measuring both goal-directed and outcome-general behavior translationally.

PIT can be studied in both humans and non-human animals, but the procedures differ between species, potentially limiting translatability. Improving comparability across animal models would provide greater insight into the fundamental mechanisms underpinning PIT that are important for motivation, decision-making, and psychopathology (Barron et al., 2020; Robinson et al., 2019). A key procedural difference between human and rodent PIT studies concerns the sensory modality of Pavlovian cues. Human PIT paradigms predominantly employ visual stimuli (e.g., Cartoni et al., 2015; Verhoeven et al., 2018), whereas rodent studies typically rely on auditory cues (e.g., Cartoni et al., 2016; Corbit et al., 2016). In rodents, this choice is intentional, as visual cues can elicit Pavlovian conditioned approach (PCA; Corbit and Balleine, 2005). Although PCA has also been demonstrated in humans, most commonly indexed as attentional capture by reward-associated visual stimuli (Bourgeois et al., 2018), it has received comparatively little consideration. Accordingly, only a small number of human PIT studies have used exclusively auditory cues (e.g., Colagiuri and Lovibond, 2015). This relative lack of concern likely reflects that human participants do not physically approach the reward site, thereby reducing the practical relevance of this potential confound.

Evidence suggests that visual cues are more salient and more readily associated with reward in humans (Meemken and Horstmann, 2019). Therefore, the use of different stimulus modalities in humans remains a potential moderator of PIT. Directly comparing auditory and visual cue modalities within the same human PIT procedure would therefore help determine whether sensory modality influences PIT performance and, critically, would strengthen the translational bridge to rodent research for future work investigating cross-species validity. This is particularly pertinent as PIT is a useful tool for measuring motivational responding under diverse conditions, such as stress and fear (Garofalo and Robbins, 2017; Pool et al., 2015).

Stress promotes automatic, general motivationally invigorated behavior in both animals and humans (Schwabe and Wolf, 2009; Wirz et al., 2018), modulating the balance between the goal-directed and habitual memory systems and favoring the latter under stressful contexts. This shift of cognitive to habitual systems under stress was illustrated by Kim et al. (2001) using a cued-water maze task, where stressed rats relied more on automatic stimulus–response systems than flexible navigation systems, compared to unstressed rats. Similar results were seen in human spatial learning tasks (Schwabe et al., 2007) and instrumental tasks in both rodents and humans (Jean-Richard-dit-Bressel et al., 2019; Ossewaarde et al., 2011; Porcelli et al., 2012; Thorsell et al., 2000). Neurally, the induction of stress results in a shift from hippocampal to dorsal striatal activation, as measured using fMRI (Braun et al., 2021; Burgess et al., 2002; Schwabe et al., 2013; Schwabe and Wolf, 2012; Seehagen et al., 2015). This is likely due to the influence of stress-related neurotransmitters and hormones, including noradrenaline and glucocorticoids (Schwabe et al., 2007; Wirz et al., 2018).

Although the impact of stress on automatic behavior converges between humans and animals, the methods used to induce stress vary markedly between the two species. In humans, procedures such as cold pressor tests, Trier Social Stress Test, and stress tests including aversive noises, aversive videos, and imagery are typically used (e.g., Cuthbert et al., 1996; Duncko et al., 2009; Grillon, 2008; Gross and Levenson, 1995; Kirschbaum et al., 1993; Neumann and Waters, 2006; Schwabe and Wolf, 2010). In animals, methods such as immobilization, footshock, forced swimming, and aversive sounds are commonly utilized to induce stress (e.g., Heinrichs and Koob, 2006; Patterson and Abizaid, 2013). To increase the immersiveness of stress in humans, ecological validity, and (perceived) uncontrollability of stressors, fully immersive threatening scenarios in virtual reality (VR) have been developed (Daniel-Watanabe et al., 2025; Quezada-Scholz et al., 2022). VR environments are powerful tools that allow a realistic, multisensory presentation of stimuli (Kothgassner and Felnhofer, 2020; Parsons, 2015) and are compatible with real-time physiological measurements of stress (Finseth et al., 2018; Krupić et al., 2021).

While utilizing VR in experimental design is promising for increasing real-world validity, it may also alter learning processes and performance in substantive ways. The immersive and all-consuming nature of these experiences could lead to changes in attention, presence, arousal, and motor output (Lawson et al., 2024; Maymon et al., 2023; Parsons, 2015). Given this, it is necessary to investigate how implementing validated procedures, such as the PIT design, in VR could modulate performance and the extent to which this could affect the measured behavioral outcomes. For example, VR introduces several sensorimotor demands, as a participant is fully immersed in a headset and will therefore engage with a task differently compared to a traditional two-dimensional (2D) computer-based design. Therefore, these novel techniques require specific investigation to understand how commonly observed behaviors are altered when a task is implemented in a different format.

This series of studies aimed to bridge the translational gap between rodent and human research investigating the impact of stress on instrumental behavior, using VR stressors to evoke either general stress and anxiety or task-relevant stress in an increasingly immersive manner. In three separate experiments, we set out to: (i) validate our PIT procedure and test whether the modality of stimulus presentation would affect the magnitude of PIT effects; (ii) compare the magnitude of PIT effects observed in positive reinforcment and negative reinforcement versions of the PIT task; and (iii) determine the impact of VR-presented stressors on the magnitude of PIT effects in individuals stratified for their level of contamination fears (CFs).

Across all three experiments, specific and general PIT effects were the primary outcomes, operationalized as baseline-corrected key-button responses during the transfer phase. Specific PIT was captured by comparing responses to outcome-congruent vs. incongruent cues. General PIT was captured by comparing responses to rewarded vs. unrewarded cues. It was predicted that specific PIT would remain robust across all conditions, reflecting stable goal-directed responding. In contrast, general PIT would be more sensitive to motivational and contextual manipulations. This led to the following specific predictions: (i) General PIT will be enhanced in immersive VR contexts relative to the 2D task due to increased arousal and motivational invigoration, while specific PIT will remain stable across experiments. (ii) Aversive contexts will increase general PIT responding due to previous accounts of stress-induced outcome-general invigoration.

## 2 Materials and methods

**Figure 1:**
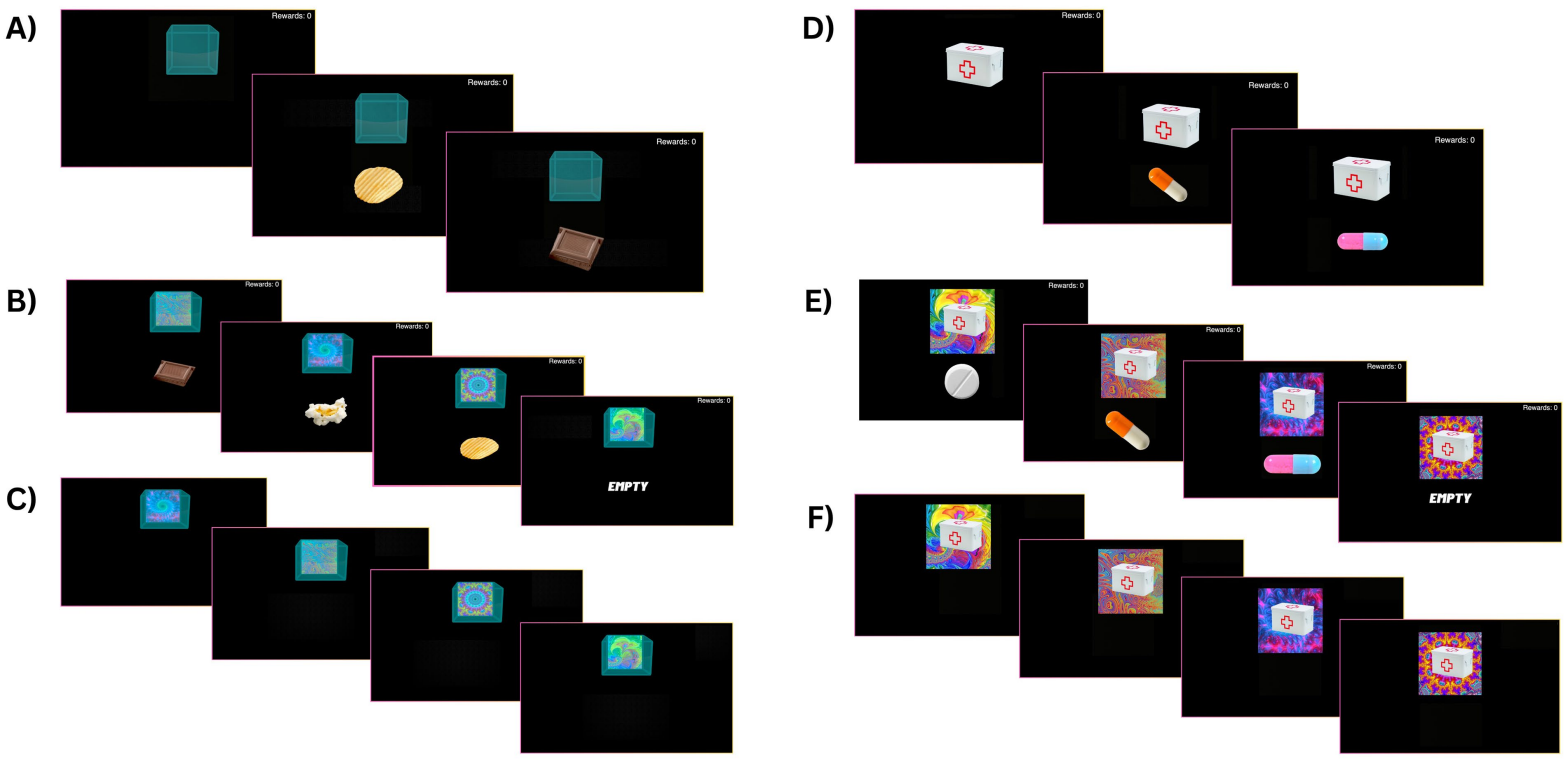
Visual version of the PIT task. **(A–C)** Appetitive task and **(D–F)** aversive task. The auditory task used version shown in images **(A–C)**; however, fractal images were replaced with four tones. **(A,D)** Instrumental phase of the PIT task, where participants respond with the left or right key (R_1_ or R_2_; see Table 1) for two rewarding outcomes (Os) from a magic or medical box. **(B,E)** Pavlovian phase of the PIT task, where four CSs are presented with four different Os. **(C,F)** Transfer phase of the visual PIT task, where the four CSs are presented in extinction; however, participants can respond with the left or right key (R_1_ or R_2_).

**Figure 2:**
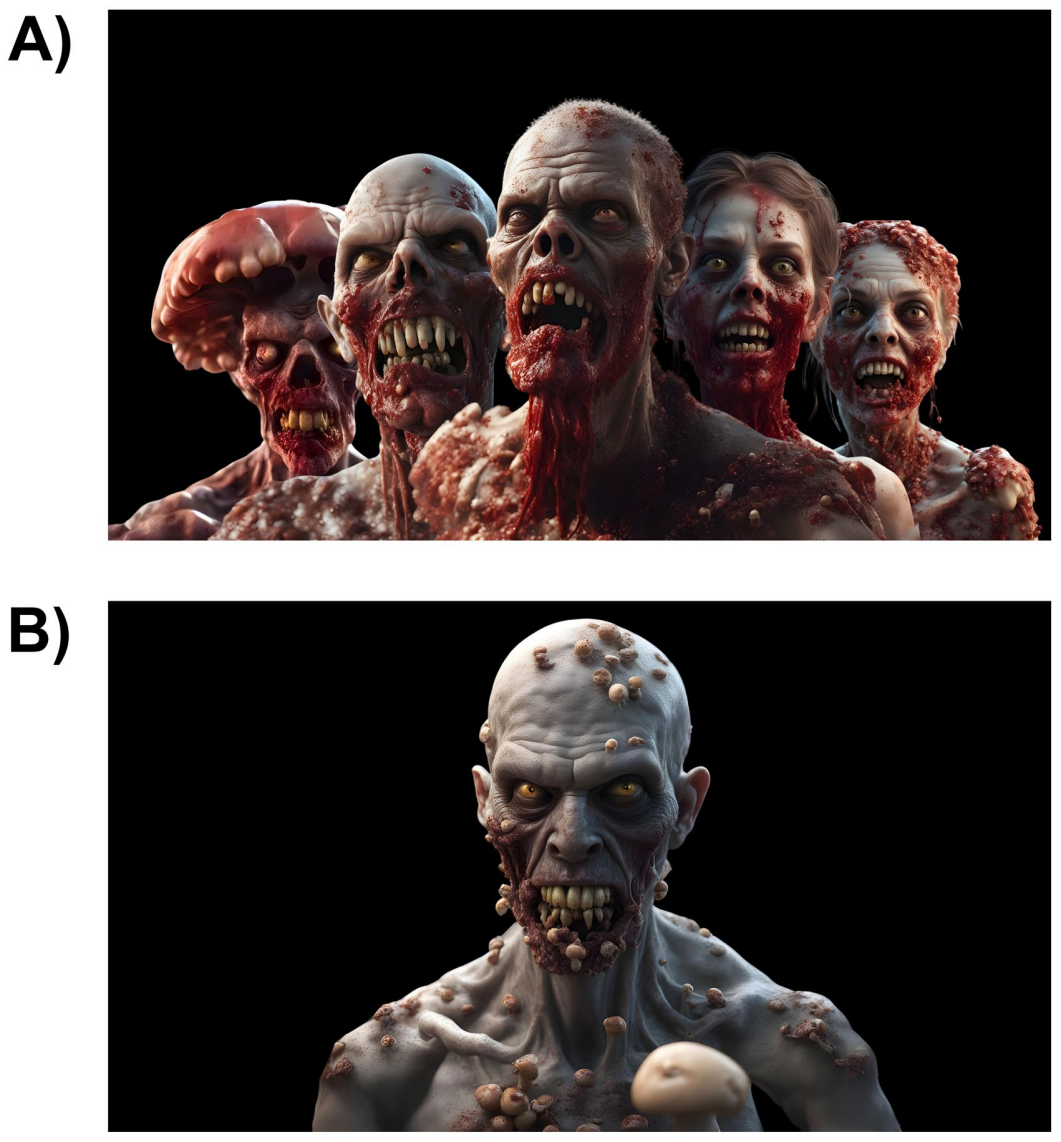
Examples of the zombie images administered to the participants as part of the instructions in Experiments 2 and 3. The skeletons were generated by www.deepai.org and edited and illustrated in Adobe Photoshop (Adobe Inc., San Jose, CA). **(A)** An example of the zombie that participants are told has infected them. **(B)** An example of the zombie that participants are told they will turn into if they do nothing in the task.

**Figure 3:**
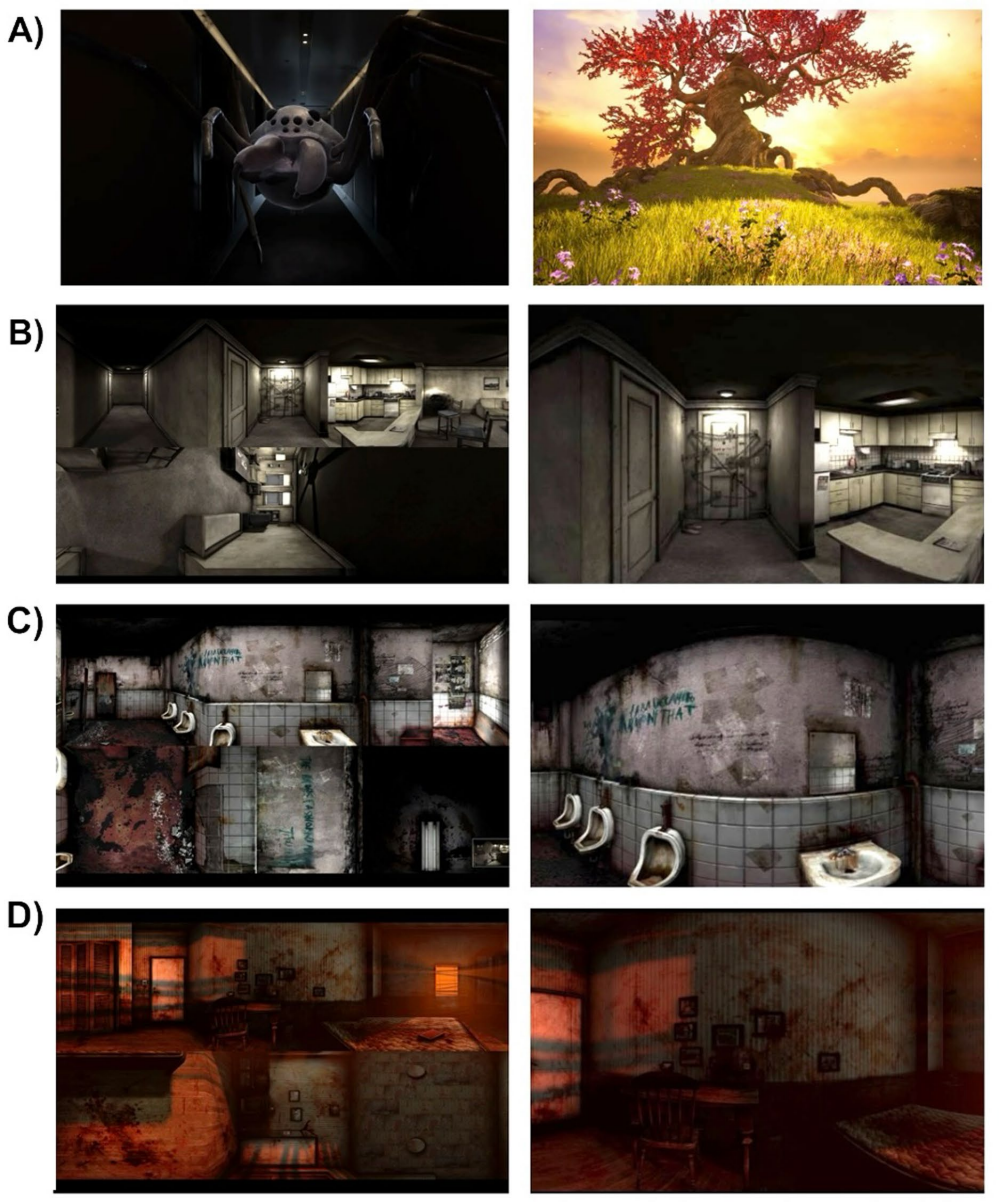
Screenshots **(A)** depict the spiders and neutral scenarios created by graphic designers in two-dimensional format at Ninja Theory Limited, which involved spiders crawling on walls (left) or a cherry blossom tree in a field (right). Screenshots **(B)** to depict a contaminated apartment, **(C)** a contaminated bathroom, and **(D)** a contaminated bedroom as examples of the contamination scenario (Project Ravenlight) in 4K equirectangular (left) and two-dimensional (right) format. These contamination scenarios were stitched in Final Cut Pro for a seamless VR experience.

A two-dimensional, computer-based PIT task was developed for administration to human participants. For Experiment 1 (Figure 1), a positive reinforcement task in which participants worked for snack rewards was developed, aligning with rodent reinforcers. Cues were presented in either auditory or visual modalities (*n* = 30 visual condition; *n* = 30 auditory condition). For Experiment 2 (Figures 1, 2), the task was adapted for administration in a VR setting and used negative reinforcement (*n* = 20) and positive reinforcement (*n* = 20). The negative reinforcement PIT task was subsequently administered in a large-scale study in people with high and low contamination fears (CFs) with a factorial design (*n* = 48 high CF; *n* = 48 low CF; Experiment 3) where state-stress was induced using immersive and realistic horror scenarios that were general (spiders; Figure 3A (left)), neutral (Figure 3A (right)) specific (contamination; Figures 3B-C) to the participants. This resulted in a general threat condition (spiders, with high CF *n* = 16; and low CF *n* = 16); *n* = 32 contamination-specific threat condition (contamination, with high CF *n* = 16; and low CF *n* = 16); and *n* = 32 neutral threat condition (neutral, with high CF *n* = 16; and low CF *n* = 16) in VR (Ninja Theory and Project Ravenlight). In Experiment 3, complementary biomarker methodologies were used, including photoplethysmography (PPG) to measure heart rate variability (HRV) and salivary alpha-amylase (sAA) to assess the extent of stress induction.

### 2.1 Participants

Across the three experiments, 196 participants were recruited via opportunity sampling using online recruitment platforms and matched within each condition by age and gender (62% female; age range 18–57 years; *M* = 22.85, standard deviation [*SD*] = 5.62). Participants in Experiment 1 (auditory vs. visual PIT; *n* = 60) were aged 18–32 years (*M* = 21.50, *SD* = 2.93; 68% female). Participants in Experiment 2 (appetitive vs. aversive context PIT; *n* = 40) were aged 18–39 years (*M* = 23.00, *SD* = 5.85; 70% female). Participants in Experiment 3 (threat × contamination fear PIT; *n* = 96) were aged 18–57 years (*M* = 23.63, *SD* = 6.61; 55% female). Within each experiment, *t*-tests confirmed that the groups were matched for age and gender, with no significant between-group differences (all *p*-values > 0.50). In Experiment 3, the Padua Inventory Contamination Fear Subscale (PI CF; Sanavio, 1988) was administered in advance and determined high (≥9) and low CF (<9) groups (according to the criteria applied by Jalal et al., 2018). In practice, the high CF group had a mean of 16.5 (*SD* = 6.89, standard error (*SE*) = 0.99, Range = 9–36), whereas the low CF group had a mean of 3.08 (*SD* = 2.45, *SE* = 0.35, Range = 0–8). Informed consent and a detailed debrief were administered for all participants.

Sample sizes were determined using *a priori* power analysis from G*Power version 3.1.9.7 (Faul et al., 2007). For the participant sample in Experiment 1, a medium-large effect size based on previous measures of specific and general PIT in humans was estimated, with *α* = 0.05 and a power = 0.80 (based on Quail et al., 2017b; *η*_p_^2^ ≥ 0.16). This indicated a minimum total sample size of *N* = 24. For Experiment 2, previous studies observed medium effect sizes using appetitive procedures [*η*_p_^2^ = 0.12 (specific PIT) and *η*_p_^2^ = 0.08 (general PIT)] and a large effect size found in PIT studies using aversive procedures [*η*_p_^2^ = 0.12 (specific PIT) and *η*_p_^2^ = 0.51 (general PIT); Garofalo and Robbins, 2017]. To achieve a medium-sized effect of *f* = 0.25, with a significance criterion of α = 0.05 and a power = 0.80 to reject the null hypothesis, the minimum sample required was *N* = 24. Furthermore, for Experiment 3, to achieve a medium effect size of *f* = 0.25 for multiple interactions (Threat × CF × Transfer) with a significance criterion of α = 0.05 and power = 0.95, the minimum required sample to reject the null hypothesis was *N* = 96. Therefore, our sample sizes exceeded the necessary sizes for power. No participants reported any cybersickness or requested to stop early. A safety protocol was in place to address any adverse effects or medical emergencies, and is described in the Supplementary material.

### 2.2 PIT tasks

The PIT task was programmed using PsychoPy software version 2021.1.4 (Peirce et al., 2019) and adapted from a procedure by Quail et al. (2017a,b) (Table 1). Code for the task and data preprocessing is available upon request from the authors. Participants were required to respond using the “*Q*” and” *P*” keys on a keyboard and to use the “*B*” key to bank rewards. Visual cues were four different fractal images, matched for size, color composition, and luminance (Figure 1). Auditory cues consisted of four different neutral tones, matched for pitch, duration, and volume, delivered centrally through a computer speaker. The rewards were three different types of foods (crisps, chocolate, and popcorn; Figures 1A–C) or three different colored pills to cure oneself from a fictional zombie virus (pink, white, and orange; Figures 1D–F). All images presented throughout the task had a resolution of 2,048–2,048 pixels, and participants were seated approximately 50 cm from the monitor. Stimuli and responses in each condition were counterbalanced. The auditory and visual appetitive PIT procedures were used in Experiment 1, and the visual aversive (zombie) PIT procedure was adopted in Experiments 2 and 3. Furthermore, in Experiments 2 and 3, this task was administered in VR using Vive Pro 2 VR equipment and a keyboard with textured Velcro buttons for responding (‘*Q*’, ‘*P*’, and ‘*B*’).

**Table 1:** Design of the PIT task used in the experiment with hypothesized outcomes for the transfer phase.

Instrumental phase	Pavlovian phase	Transfer
R_1_ → O_1_	CS_1_ → O_1_	CS_1_ → R_1_ > R_2_
R_2_ → O_2_	CS_2_ → O_2_	CS_2_ → R_2_ > R_1_
	CS_3_ → O_3_	CS_3_ → R_1_ or R_2_
	CS_4_ → O_4_ (EMPTY)	CS_4_ → No R

Adapted from Degni et al. (2022).

R, response; O, outcome; CS, conditioned stimuli.

#### 2.2.1 Task framing

In the appetitive versions of the task, participants began by rating how much they liked popcorn, crisps, and chocolate and how hungry they were on a scale of 0–7. In the aversive version, participants were told a fictional zombie virus had infected them (and presented with the images in Figures 2A,B) and that they had to collect different pills to cure themselves from it, but were not told which pill would work (Figure 1D; O_1_, O_2_, and O_3_; pink, white, or orange pills). Full instructions for both tasks are available in the Supplementary material. The three phases of the PIT task were presented sequentially for all participants, with no breaks: instrumental followed by Pavlovian, and then the transfer test.

#### 2.2.2 Instrumental phase

For the instrumental phase in all versions of the task (Figures 1A,D), the participants were verbally instructed to respond by pressing one of two keys (“*Q*” and “*P”*) with their index fingers to tilt the box left or right (R_1_ and R_2_) to receive a reward (O_1_ and O_2_; two of chocolate, crisps, and popcorn in Experiment 1 or two of a pink, white or orange pill in Experiments 2 and 3) with no third outcome available. After each response, the box immediately tilted left or right before returning to its original vertical position after 1 s. Rewards were delivered according to a variable-ratio schedule, whereby a response requirement was randomly sampled between 5 and 9, and participants were required to continue responding on the same side until this threshold was exceeded to obtain the reward. After an image of the reward appeared on the screen, participants had 1 s to press “*B*” to bank the reward they had earned. The total was then tallied and displayed in the top-right corner of the screen before the reward disappeared. The amount of banking did not influence task progression but was implemented to make the procedure more realistic and engaging and to align with the consummatory response observed in rodent instrumental procedures. However, this banking response was not an explicit attention check; the multiple-choice questions in the instrumental and Pavlovian phases served this purpose instead. Instrumental training ended once 18 rewards were acquired. To check that participants were learning, questions on reward contingency were presented after the delivery of every third reward, where participants would be presented with a picture randomly assigned of O_1_ or O_2,_ and asked to indicate if they pressed the “*left*” or “r*ight*” button, and participants had no deadline for responding to the multiple-choice questions. This phase took approximately 5 min to complete.

#### 2.2.3 Pavlovian phase

During the Pavlovian-conditioning phase (Figures 1B,E), cues (visual or auditory in Experiment 1, Figure 1B) were associated with three food rewards in the appetitive version or three different pills in the aversive version (Experiments 2 and 3, Figure 1E), two of which had been experienced during the prior instrumental phase (O_1_ and O_2_) and an additional third unique outcome (O_3_). A fourth cue was associated with no reward and the display of the word “*EMPTY*” on the screen. Participants were told to watch the box (or listen while watching it in the auditory condition) and to press the key “*B*” to collect rewards.

Cues were presented for 3 s, and the rewards were displayed underneath the box for 2 s, 1 s after the cue was presented, with an inter-trial interval (ITI) of 5 ± 1 s. Each of the 4 cue-outcome pairings (see Table 1) were presented 12 times for a total of 48 trials. Participants needed to press “*B*” to bank the reward during its 2-s presentation. Banking had no influence on task progression in the Pavlovian phase. A question after each of the cue-outcome pairings (four reward presentations) assessed the learning contingencies where participants would be randomly presented with one of the four cues and asked to indicate if it was associated with “*(a) crisps, (b) chocolate, (c) popcorn, or (d) empty*” in the appetitive versions and “*(a) pink, (b) orange, (c) white, or (d) empty*” in the aversive version. The order of the reward options was also randomized for each question presentation, and the questions remained on screen until a response was provided. The questions ascertained the extent to which the participant correctly learned the associations. This phase took approximately 10 min.

#### 2.2.4 Transfer phase

In the transfer phase, participants were instructed to use the left and right buttons again to get the rewards to fall out of the box; however, they were told that the rewards were now invisible, and they no longer had to bank them (Figure 1C for snack rewards in the appetitive version and Figure 1F for pill rewards in the aversive version). The cues presented in the transfer phase were presented for 3 s, 14 times each, with an ITI of 5 ± 1 s (for a total of 56 trials). No rewards were delivered in this phase. This phase lasted approximately 10 min.

### 2.3 VR state-stress scenarios

In Experiment 3, VR compound scenarios were developed employing distinct sounds, smells, and visuals. For each participant, one of the three scenarios was presented, and immediately following this, the PIT task began within the headset (approximately 30 s from the end of the scenario). Two scenarios were crafted by Ninja Theory Limited (Figure 3A): a threatening scenario where spiders would crawl over the walls and onto the participants, or a neutral scenario where participants would sit in front of a cherry blossom tree. These have been specifically designed to evoke different emotional responses (Daniel-Watanabe et al., 2025), found to be well tolerated, and have not had any adverse events to date. An additional contamination scenario was created by Project Ravenlight (Figures 3B–D); a contamination threatening one where participants would move through various contaminated rooms, including a bathroom, kitchen, and living room. Participants could terminate or pause the experiment at any time, but none did so. In addition to the VR scenarios, to enhance state-stress, a diffuser emitted a “dungeon” scent (AromaPrime, Rochdale, UK) and an ominous soundtrack played in the background throughout the entirety of the experiment in the contamination and spider conditions, including during cognitive tasks. In the neutral condition, there was an ambient focus background soundtrack and no diffuser scent. In Experiment 3, PPG measures, sAA biomarkers, and Likert scales [including the State Anxiety Scale (STAI-S) (Spielberger et al., 1983) and scales asking participants how “scared” and how “disgusted” they were on a scale of 0–10] were collected at both pre- and post-test with the sAA sampling after the STAI-S at pre-test and before the STAI-S at post-test, to validate the effect of stress throughout the experimental conditions and at pre- and post-test. For detailed biomarker methods and background sound stimuli (see the Supplementary material).

### 2.4 PIT analyses

Analyses were performed in Python (Spyder 6.0.3). Specific and general PIT were derived from baseline-corrected response rates [Experiment 1: 2 × 2 analysis of variance (ANOVA) (Modality × Transfer Type); Experiment 2: 2 × 2 ANOVA (Context × Transfer Type); Experiment 3: 2 × 2 × 3 ANOVA (Contamination Fear Level × Scenario × Transfer Type)]. Bayesian model comparisons were conducted to test for null effects. Participants were required to reach ≥ 80% (instrumental) and ≥ 75% (Pavlovian) contingency accuracy; non-learners were excluded [Experiment 1 *N* = 12 (*n* = 6 in auditory and *n* = 6 in visual modality groups); Experiment 2 *N* = 6 (*n* = 4 participants in the appetitive and *n* = 2 participants in the aversive phases); Experiment 3 *N* = 9 (*n* = 5 with high CF and *n* = 4 with low CF)]. In addition, generalized linear models (GLMs) were implemented to examine the effects of VR condition, stress, and context on specific and general PIT outcomes across all visual conditions from the above experiments (*N* = 196). Detailed statistical analyses for all elements of this section are available in the Supplementary material. All statistical analysis code for this study is available on request.

### 2.5 PPG and sAA analyses

The PPG data (75 Hz) (Experiment 3) were pre-processed in Python (using the Systole package in version 6.0.3 of Python) and MATLAB (R2024b). Data were segmented into task phases (baseline, scenario, instrumental, Pavlovian, and transfer phases) and filtered with a 0.5–8 Hz Butterworth bandpass filter. Mean, median, max heart rate (HR), and standard deviation of normal-to-normal intervals (SDNN) were computed and used as a state-stress index (Shaffer and Ginsberg, 2017) (see Supplementary material for details). Mixed factorial ANOVAs tested main/interaction effects of the coefficient of variation of SDNN (cvSDNN) by experimental phase, contamination fear, and threat group. sAA (Experiment 3) was corrected for flow rate, where participants were told not to eat 8 h before the experiment, and sampling times were immediately before the experiment, when they entered the room, and immediately after the PIT task, and were tested between 10 a.m. and 4 p.m. (see Supplementary material for details). Mean pre- and post-test sAA levels (U/min) were compared using 2 × 2 × 3 ANOVAs (time × CF group × scenario).

## 3 Results

### 3.1 Robust PIT effects were observed across all conditions and experiments

The PIT effects were calculated by first computing baseline responding (key presses per second outside cue presentation), which was subtracted from responding during the cues. Specific PIT was calculated as the difference between congruent and incongruent response rates (R_1_ and R_2_ summed within outcome and averaged across trials). In contrast, general PIT was computed as the difference in response rates between CS_3_ and CS_4_, with no exclusion of negative values after baseline correction, as they reflect meaningful reductions in responding relative to baseline. Baseline responding rates during the ITI were subtracted from the cue response rates (see Table 2 and Supplementary material for further information and more detailed statistics).

**Table 2:** Responding during PIT tasks across experiments by CS minus baseline ITI responding and specific and general PIT.

Experiment	Condition	Measure	Mean	SD	*N*
1	Auditory	CS Same	2.35	1.95	30
		CS Different	−0.25	0.75	30
		CS_3_	1.96	1.62	30
		CS_4_	0.05	0.88	30
		Specific PIT	1.44	2.26	30
		General PIT	0.59	2.90	30
1	Visual	CS Same	2.47	1.72	30
		CS Different	−0.24	0.87	30
		CS_3_	2.41	1.60	30
		CS_4_	0.26	1.38	30
		Specific PIT	2.26	1.77	30
		General PIT	1.49	2.10	30
2	Appetitive	CS Same	2.63	1.06	20
		CS Different	0.23	0.67	20
		CS_3_	2.50	1.38	20
		CS_4_	−0.15	0.12	20
		Specific PIT	2.21	1.52	20
		General PIT	2.46	1.37	20
2	Aversive	CS Same	2.50	1.36	20
		CS Different	0.53	1.19	20
		CS_3_	2.27	1.53	20
		CS_4_	0.20	0.78	20
		Specific PIT	1.75	2.41	20
		General PIT	1.86	1.71	20
3	Neutral	CS Same	2.68	1.10	32
		CS Different	0.32	0.89	32
		CS_3_	2.77	1.55	32
		CS_4_	−0.07	0.60	32
		Specific PIT	2.07	1.77	32
		General PIT	2.55	1.64	32
3	Spiders	CS Same	2.16	1.38	32
		CS Different	0.71	1.20	32
		CS_3_	2.85	1.23	32
		CS_4_	−0.10	0.54	32
		Specific PIT	1.06	2.38	32
		General PIT	2.56	1.42	32
3	Contamination	CS Same	2.38	1.45	32
		CS Different	0.55	0.95	32
		CS_3_	2.73	1.64	32
		CS_4_	−0.04	0.67	32
		Specific PIT	1.55	2.27	32
		General PIT	2.47	1.80	32

For Experiment 1, PIT was observed in both the auditory and visual modalities with large effects for both general cue responding [*F*_(1, 58)_ = 89.80, *p* < 0.001, *η*_p_^2^ = 0.61; *M_diff_* = 2.03, 95% CI (1.60, 2.46)] and specific cue responding [*F*_(1, 58)_ = 124, *p* < 0.001, *η*_p_^2^ = 0.68; *M_diff_* = 2.66, 95% CI (2.18, 3.14)] (see Supplementary material for detailed calculations). Numerically, responding was higher in the visual condition, but this did not reach statistical significance (*p* > 0.05). Specific PIT effects were greater than general PIT effects across both conditions [Type of Transfer: *F*_(1, 58)_ = 6.81, *p* = 0.01, *η*_p_^2^ = 0.03; Type of Transfer × Modality: *F* < 1], with a small difference between general and specific PIT [*M_diff_* = −0.81, 95% CI (−1.44, −0.19)]. Follow-up Bayesian model comparisons provided evidence for the absence of the Transfer Type × Modality interaction [BF_01_ = 7.69] with evidence that general [BF_01_ = 6.61] and specific PIT [BF_01_ = 7.50] did not differ across modalities.

In Experiment 2, PIT effects were observed in both the positive reinforcement (snack) and negative reinforcement (zombie) versions of the task, with similar effects across the two contexts [*F*_(1, 38)_ = 1.29, *p* = 0.26] and large effects for both general cue responding [*F*_(1, 38)_ = 85.89, *p* < 0.001, *η*_p_^2^ = 0.69; *M_diff_* = 2.36, 95% CI (1.84, 2.87)] and specific cue responding [_*F*(1, 38)_ = 45.94*, p* < 0.001, *η*_p_^2^ = 0.55; *M_diff_* = 2.18, 95% CI (1.54, 2.83)]. However, there were no significant differences between the snack or zombie versions of the task for both general or specific PIT [Context × Transfer Type: *F*_(1, 38)_ = 0.06, *p* = 0.81, *η*_p_^2^ = 0.002]; with no difference between general and specific PIT [*M_diff_* = 0.18, 95% CI (−0.48, 0.83)]. Follow-up Bayesian model comparisons provided evidence for the absence of the Transfer Type × Context interaction [BF_01_ = 6.13] with general [BF_01_ = 3.21] and specific PIT [BF_01_ = 5.03] by context.

Similarly in Experiment 3, as for Experiment 2, administration of the task in the VR headset led to greater levels of general PIT, compared to specific PIT [Transfer Type: *F*_(1, 90)_ = 12.8, *p* < 0.001, *η*_p_^2^ = 0.12; *M_diff_ =* 0.97, 95% CI (0.43, 1.51)], with large effects for both general cue responding [*F*_(1, 90)_ = 279.18, *p <* 0.001, *η*_p_^2^ = 0.76; *M_diff_* = 2.85, 95% CI (2.51, 3.19)] and specific cue responding [*F*_(1, 90)_ = 72.68, *p <* 0.001, *η*_p_^2^ = 0.45; *M_diff_* = 1.88, 95% CI (1.44, 2.32)] (see Supplementary material for detailed calculations). Contrary to expectations, there were no differences in PIT between participants who had experienced the neutral condition, general spider stress or contamination-specific stress [Threat Group: *F*_(2, 90)_ = 1.01, *p* = 0.37], even when self-reported contamination fears were used to split the group [CF: *F* < 1; CF × Threat Group: *F* < 1]. Neither the type of threat experienced, nor self-reported contamination fears altered the extent of transfer effects [Transfer Type × Threat Group: *F*_(2, 90)_ = 1.18, *p* = 0.31; Transfer Type × CF: *F* < 1; Transfer Type × CF × Threat Group: *F* < 1]. Bayesian model comparisons further provided strong evidence against this three-way interaction [Transfer Type × CF × Threat Group: BF_01_ = 89.73], which persisted for specific PIT [BF_01_ = 78.29] and general PIT [BF_01_ = 55.44] with respect to CF and Threat Group.

### 3.2 Physiological and self-report stress measures in Experiment 3

#### 3.2.1 Heart rate variability (HRV)

**Figure 4:**
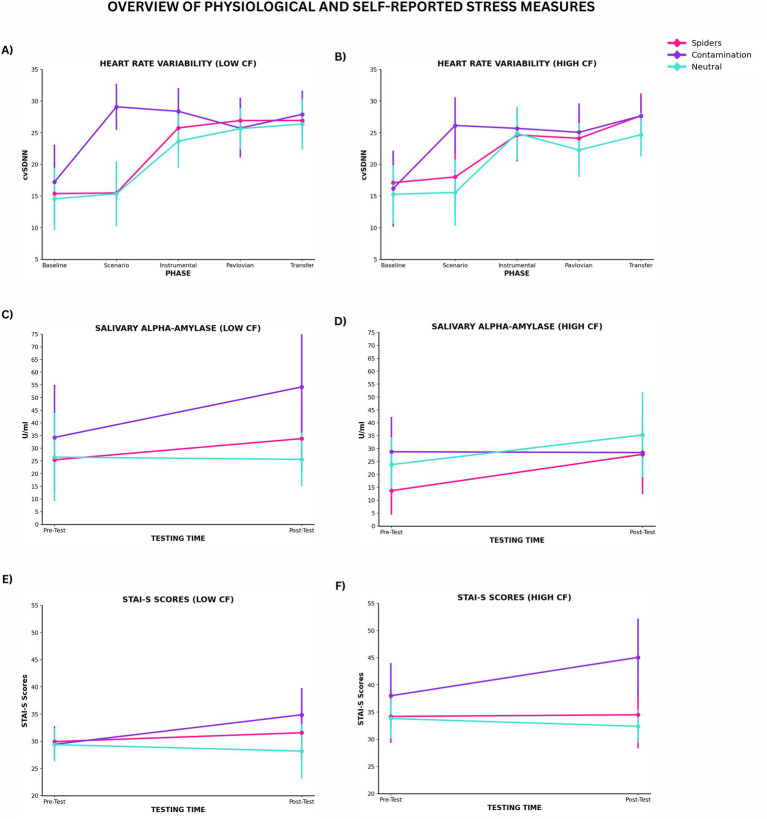
Overview of physiological and self-reported stress measures in Experiment 3, including HRV across the experimental phases for low **(A)** and high CF **(B)**, sAA at pre- and post-test for low **(C)** and high CF **(D)**, STAI-S scores at pre- and post-test for low **(E)** and high CF **(F)**. Bars represent 95% confidence intervals.

In Experiment 3, to measure state-stress, HRV was collected through PPG (Figures 4A,B). These measures were compared across experimental phases, threat scenario groups, and CF groups. A 5 × 3 × 2 mixed measures ANOVA was administered to compare the coefficient of variation of the SDNN (cvSDNN) across experimental phases (baseline, scenario, instrumental, Pavlovian, and transfer phases) and CF (high and low) and threat group (spiders, contamination, and neutral) as a measure of HRV.

The greatest increases in HRV were observed during the experimental phase [Phase: *F*_(4, 360)_ = 52.7, *p* < 0.001, *η*_p_^2^ = 0.37] and differed across experimental conditions [Threat Group: *F*_(2, 90)_ = 3.66, *p* = 0.03, *η*_p_^2^ = 0.08], with the greatest increases in HRV being observed in the contamination group [Phase × Threat Group: *F*_(8, 360)_ = 5.93, *p* < 0.001, *η*_p_^2^ = 0.12]. *Post hoc* comparisons using Holm’s method indicated that while the contamination-specific group showed greater HRV changes than both the neutral [*M* = 6.98, *SE* = 2.09, *t*(62) = 3.33, *p_holm_* = 0.004] and the spider stress groups [*M* = −5.67, *SE* = 2.09, *t*(62) = −2.71, *p_holm_* = 0.02], the neutral and spider stress groups did not differ from each other [*M* = 1.31, *SE* = 2.09, *t*(62) = 0.63, *p_holm_* = 0.53]. However, self-reported contamination fears did not affect HRV [CF: *F* < 1; Phase × CF: *F* < 1], even when high CF participants were exposed to contamination-specific stressors [CF × Threat Group: *F* < 1; Phase × CF × Threat Group: *F* < 1].

#### 3.2.2 Salivary alpha-amylase (sAA)

Samples of sAA were collected before and after the experimental session to capture state-stress differences (Figures 4C,D). A 3 × 2 mixed measures ANOVA was administered to compare the sAA across the pre-test and post-test (testing time) sampling sessions and CF (high and low) and threat group (spiders, contamination, and neutral). sAA levels were higher overall at post-test [sAA Testing Time: *F*_(1, 90)_ = 10.9, *p* < 0.001, *η*_p_^2^ = 0.12], with the greatest increases observed in the low CF participants experiencing the contamination scenario [sAA Testing Time × Threat Group × CF: *F*_(2, 90)_ = 3.51, *p* = 0.03, *η*_p_^2^ = 0.07]. Self-reported contamination fears had no overall effect on sAA changes [CF: *F*_(1, 90)_ = 1.45, *p* = 0.23; sAA Testing Time × CF: *F* < 1; CF × Threat Group: *F* < 1], and there were no overall differences observed between participants in different experimental conditions [Threat Group: *F*_(2, 90)_ = 1.37, *p* = 0.26; sAA Testing Time × Threat Group: *F* < 1].

#### 3.2.3 Self-report measures

Subjective self-report measures using the STAI-S and 0–10-point Likert scales were administered pre- and post-test to examine self-reported fear and disgust at each time point (Figures 4E,F). Participants in all groups reported greater state anxiety post-test [STAI-S: *F*_(1, 90)_ = 5.15, *p* = 0.03, *η*_p_^2^ = 0.05], with those in the threat groups [STAI-S × Threat Group: *F*_(2, 90)_ = 6.66, *p* = 0.002, *η*_p_^2^ = 0.13] and participants with high CF [CF: *F*_(1, 90)_ = 13.1, *p* < 0.001, *η*_p_^2^ = 0.13] reporting greater increases in state anxiety, though with no interactions [CF × Threat Group: *F*_(2, 90)_ = 1.30, *p* = 0.28; STAI-S × CF: *F* < 1; STAI-S × CF × Threat Group: *F* < 1]. Participants in the high CF group had higher levels of state anxiety (STAI-S) at both pre- and post-test compared to participants in the low CF group [Pre-Test: *t* = 3.75, *d* = 0.77, *p* < 0.05; Post-Test: *t* = 2.66, *d* = 0.54, *p <* 0.05]. The high CF group participants also had significantly higher feelings of disgust and appraisals of how disgusting the contamination scenario was compared to low CF [Disgust: *t* = 3.41, *d* = 1.21, *p* = 0.002; Disgust Appraisal: *t* = 2.94, *d* = 1.04, *p* = 0.007]. All participants who experienced the contamination and spiders scenarios self-reported increased disgust and fear on a Likert scale post-test (*p* < 0.05).

### 3.3 General and specific PIT effects across experiments 1–3

**Figure 5:**
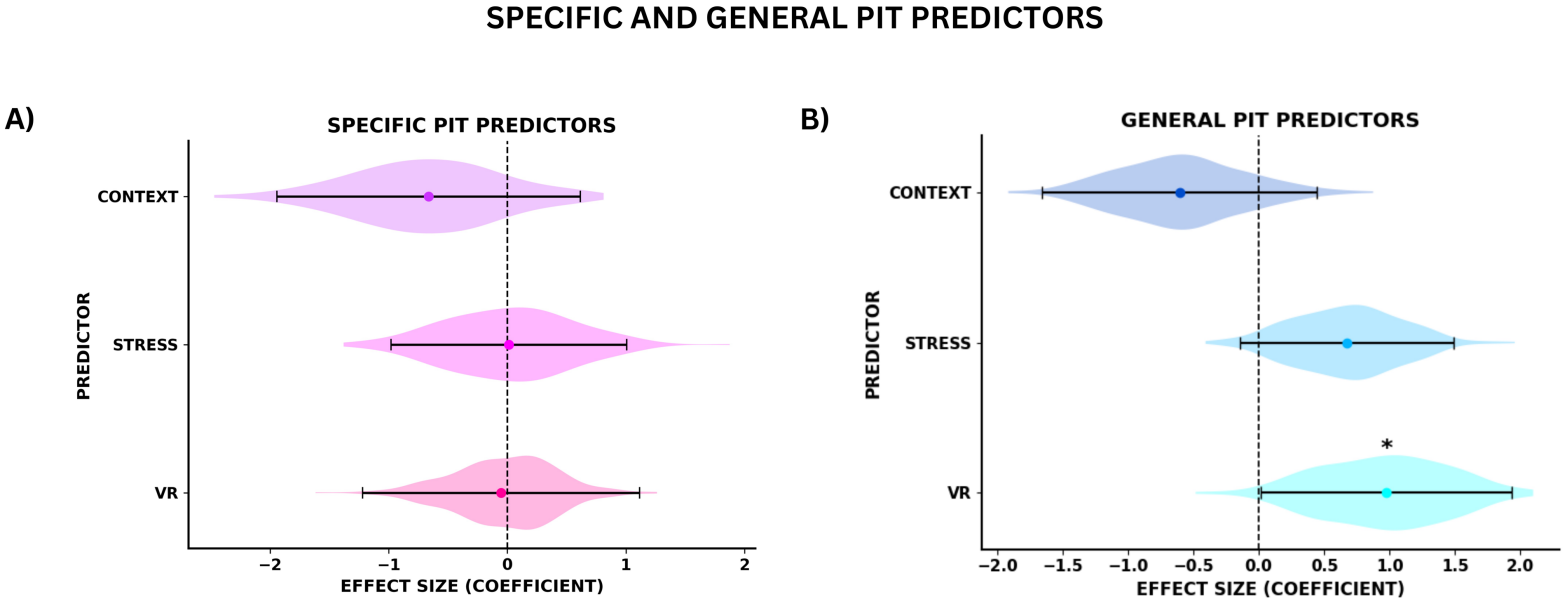
Overview of the GLM model coefficients for specific PIT **(A)** and general PIT **(B)** according to context (appetitive vs. aversive context), stress (including a stress induction scenario vs. not), and virtual reality (2D vs. 3D presentation) predictors. Violins represent bootstrapped coefficient distributions, points indicate model estimates with 95% confidence intervals, and the asterisk (*) denotes *p* < 0.05.

A general linear model (GLM) was used to examine whether presenting the PIT task in VR (compared to 2D), inducing stress (using a negatively reinforced rather than positively reinforced version of the task), and contextual threat (with relevant threatening scenarios compared to without) predicted specific and general PIT performance. Individuals in the high and low CF groups were collapsed into a single group based solely on contextual threat, given the lack of significant between-group differences in the earlier analyses (*p* > 0.05). This resulted in an analysis of 6 of 7 groups across all experiments, as the auditory group in Experiment 1 was excluded due to the difference in stimulus modality. The final groups for the GLM were the following: (1) 2D appetitive PIT, (2) three-dimensional (3D) appetitive PIT, (3) 3D aversive PIT, (4) 3D neutral threat aversive PIT, (5) 3D spiders threat aversive PIT, and (6) 3D contamination threat aversive PIT (Figure 5). For the specific PIT, the overall model was not significant (pseudo-*R*^2^ = 0.02). None of the individual predictors reached significance: VR condition: *β* = −0.05, *SE* = 0.60, *z* = −0.09, *p* = 0.928, 95% CI [−1.22, 1.11]; stress: *β* = 0.01, *SE* = 0.51, *z* = 0.02, *p* = 0.980, 95% CI [−0.98, 1.01]; and contextual threat: *β* = −0.66, *SE* = 0.65, *z* = −1.02, *p* = 0.309, 95% CI [−1.94, 0.62]. These findings show that specific PIT was not significantly modulated by VR immersion, context of the PIT task, or the introduction of contextual threat.

In contrast, for general PIT, the overall model was significant (pseudo-*R*^2^ = 0.06). The VR condition was a significant positive predictor of general PIT: *β* = 0.98, *SE* = 0.49, *z* = 1.99, *p* = 0.046, 95% CI [0.02, 1.94]. This provides evidence that presenting the PIT procedure in VR enhances the general PIT effects compared to non-VR procedures. The predictors of stress and context were not significant (stress: *β* = 0.67, *SE* = 0.42, *z* = 1.61, *p* = 0.108, 95% CI [−0.15, 1.49]; context: *β* = −0.61, *SE* = 0.54, *z* = −1.13, *p* = 0.258, 95% CI [−1.66, 0.45]).

## 4 Discussion

The current study examined general and specific PIT across three experiments that tested different tasks, reinforcements, and assessed the impact of stress on responding in healthy individuals. Despite wide variation in the utilized PIT procedures across the experiments, robust specific and general PIT effects were observed in auditory and visual modalities, with positive and negative reinforcement, and in 2D and immersive VR settings to evoke both reward-oriented and outcome-general behavior in healthy volunteers. The key finding was an indication that VR immersion may selectively amplify general transfer while sparing specific PIT. There were several procedural factors that may have influenced these results, for example, the influence of hardware differences across contexts, that is, the use of Velcro buttons in the VR conditions, potentially modulating motor ergonomics and therefore the way individuals responded to the task. As such, this interpretation should be treated cautiously. Moreover, because the GLM predictors are not orthogonal, we cannot infer causality; instead, we provide an overview of PIT effects across procedural conditions and contexts that vary in threat, immersion, and stimulus presentation. Nevertheless, this research provides evidence that a fully immersive, stressful environment can evoke strong levels of both goal-directed and outcome-general responding on an adapted and validated PIT task featuring a threatening storyline. This evokes naturally occurring behavior in a manner that could be defined as more generalizable to everyday life than artificial computer-based tasks, and provides insights into potential directions for better understanding behavioral processes in psychopathology.

Experiment 1 sought to determine whether visual and auditory stimuli would be equally effective in supporting general and specific PIT in humans. Rodent PIT procedures commonly use auditory cues to avoid the potential confound of PCA to the cue (Cartoni et al., 2016), while human studies typically use visual cues (e.g., Verhoeven et al., 2018). Any difference in PIT effects across modalities could be potentially problematic for translation across species. To date, no study has probed the role of stimulus modality on the strength of general and specific PIT effects in appetitive PIT procedures in humans. The current findings showed that specific and general PIT effects were observed regardless of whether the cues were auditory or visual. As the current study did not explicitly assess PCA or attentional capture, it cannot be concluded whether PCA-adjunct processes drive any modality-related effects. These findings instead can be interpreted as establishing that PIT is robustly elicited in both auditory and visual modalities in humans under equally controlled conditions. Further testing, possibly including eye movement assessments, is necessary to investigate whether PCA is equally present across modalities in humans and, if it exists, how it may contribute to the modulation of PIT effects.

The use of visual stimuli for the aversive contamination-based PIT task adaptation was informed by Experiment 1 and implemented in Experiments 2 and 3, alongside VR methods and sensory immersion through ambient sound and horror smells in the laboratory. There were no between-groups differences in PIT in Experiment 2 between the appetitive and aversive contexts; however, administering the PIT task in VR increased the effect size for general PIT. Though not significant, the specific PIT effect sizes decreased in VR compared to outside the immersive environment, which could be attributed to the state-stressor induced by the aversive context of the PIT task impairing goal-directed learning. Future research should probe the role of immersive stressors further and clarify their potential to influence goal-directed learning.

Consistent PIT effects were also observed across groups in Experiment 3 for threat and for individuals with high and low contamination fears (CFs), perhaps due to the fact that all groups showed increases in acute stress responses, as measured via sAA triggered by increases in noradrenaline, across the course of testing (Ali and Nater, 2020; Granger et al., 2007; Nater et al., 2006; Nater and Rohleder, 2009). Similarly, while there were differences in HRV as measured by cvSDNN across the threat scenarios and the instrumental, Pavlovian, and transfer phases, there were no differences between high- and low-CF groups. This absence of between-group differences could be attributed to the baseline phase already being particularly threatening to all participants due to the knowledge that they would be engaging in a horror task and the olfactory stimuli and auditory ominous background music that were present in the spiders and contamination scenarios, suggesting that the procedure may need to be refined to adequately probe stress effects in PIT (Hanich, 2011; Thayer and Levenson, 1983). Smell is thought to be particularly powerful when evoking emotion; the scent alone may have increased anxiety without additional intentional stressors (Ehrlichman and Bastone, 1992; Krusemark et al., 2013). Moreover, the negatively reinforced PIT task included the zombie storyline for all participants, even those in the neutral condition. The storyline behind the task could be considered aversive and fear-evoking alone. Certainly, all participants reported increased state anxiety on the STAI-S post-test, though the self-report scales indicated the contamination scenario was the most effective, with baseline stress levels being highest in the CF group, which is consistent with there being at least some level of contamination-related emotion and aversion being elicited in the current study.

It is evident that the negatively reinforced PIT task procedure elicited state-stress in participants in Experiment 3, as evidenced by increases in post-test sAA, STAI-S scores, and self-reported fear and disgust ratings. However, it is unclear which design factors were driving the rise in stress, as the current study included several novel elements and procedures. Future studies should probe these elements separately to understand their possible specific and interactive contributions to increases in state-stress.

The observed trends in HRV were less clear, consistent with the mixed literature on the effects of stress on HRV. Common observations report a decrease in HRV when stress is present in some contexts; however, this is not strictly unidirectional (Kim et al., 2018; Hildebrandt et al., 2016). Other accounts suggest that HRV can increase under stress, reflecting flexible prefrontal regulation of autonomic responses and flexible top-down control (Gonçalves et al., 2021; Hildebrandt et al., 2016). In the current study, we observed greater increases in HRV under stress conditions, which could reflect heightened attentional engagement, resulting in adaptive regulation to sustained threat and amplifying parasympathetic recruitment. This would align with the immersive, novel environment the participants experienced in the stress condition. Moreover, our sample may have, by chance, had greater overall adaptability to stress, thus reflecting the increases in HRV. This could suggest that HRV reflects more flexible autonomic responding to environmental demands, especially in unpredictable environments. However, the precise role of HRV in aversive PIT and learning tasks requires additional investigation.

Despite the lack of significant group differences in PIT and potential procedural confounds across experiments, the magnitude of PIT differed across experiments. As the context and immersive nature of the experiments grew, so did the general PIT effect, in line with its anticipated sensitivity to motivational and affective processes elicited by the Pavlovian cues. There was an increase in effect sizes observed between the two-dimensional and three-dimensional immersive VR PIT task versions, which is consistent with accounts in which uncertainty and stress shift goal-directed, outcome-specific control processes toward an overall invigoration of general responding (e.g., Garofalo and Robbins, 2017). When considering how this is captured in PIT, this is expressed through an increase in response vigor, or general PIT, in the presence of Pavlovian cues, regardless of the specific outcome. Mechanistically, stress-related arousal may enhance the motivational “energizing” impact of Pavlovian cues (e.g., via heightened salience and affective arousal), promoting stimulus–response responding that is less constrained by expected outcomes, an effect previously observed for aversive Pavlovian cues modulating instrumental behavior in humans (Garofalo and Robbins, 2017). In contrast to the changes in general PIT, specific PIT appeared consistent in magnitude across all contexts. We speculate that goal-directed learning, as measured by specific PIT, is robust against novel contexts, but in situations of uncertainty, excitement, and fear, outcome-general, more automatic pathways that are observed in habit formation become more pronounced in healthy volunteers, consistent with Schwabe and Wolf (2010). However, direct tests of this, for example, including outcome-devaluation methodology, are necessary to confirm these hypotheses (Perkes et al., 2023).

Immersion and attention were promoted through a gamified, motivating experiment featuring an engaging storyline (i.e., a fictional zombie virus). The current research also presented a PIT task that was able to integrate both robust physiological stress and behavioral measures into the same experimental setting, which could be used to explore stress and behavior in future translational cross-species tasks. Overall, this study demonstrates that PIT is a robust and reliable way of measuring stimulus-bound and goal-directed behaviors that seem to persist irrespective of immersion, threat, or procedure, and that the use of immersive, simulated threat contexts could potentially foster automatic outcome-general learning in healthy and clinical populations; however, this would require further investigation to confirm. The findings have wide-ranging theoretical and clinical implications. These include mechanistic implications, namely how VR is demonstrated to increase motivational salience of Pavlovian cues through general PIT in arousing and immersive environments. This provided evidence that this general motivational effect promotes response invigoration outside the constraints of outcome-specific representations. These insights provide support for models suggesting that heightened arousal and stress can bias behavior toward a general motivating effect underpinning disorders of maladaptive habit formation (Berg, 2025). From a clinical perspective, this aversive, immersive PIT procedure offers a novel experimental representation of cue-driven behavior, which can be utilized to investigate compulsive avoidance and threat-driven responding in conditions such as OCD and addiction through further adaptation and experimentation (Garbusow et al., 2016; Gillan et al., 2015; Hunt et al., 2022; Marzuki et al., 2024). This research provides a way to capture both physiological stress responses and associated cue-driven behavior, which could inform a framework utilized for identifying individual differences in potential maladaptive habit formation vulnerability through differences in learning styles. These immersive procedures capturing this phenomenon could help inform potential treatments and interventions for maladaptive behavior to better understand how to modulate Pavlovian influences over action in clinical contexts.

There are also methodological implications as VR can successfully increase ecological validity while retaining experimental control and biomarker acquisition. This experiment is additionally the first time that the traditional laboratory-based PIT procedure has been administered in immersive VR contexts. Therefore, refining such procedures to further uncover exactly how we learn under stress, especially when learned behavior becomes problematic and maladaptive, is an important research question that warrants further exploration.

Despite the strengths of the current multi-experiment design in innovating PIT procedures, there are several limitations that must be considered when interpreting the current findings. First, the comparison between 2D and novel VR procedures, including VR-specific confounds such as altered motor ergonomics, was not clearly isolated, and the reinforcement contexts, together with the differing levels of immersion, may covary and inflate the general PIT measures through increased vigor and excitement. Due to this, the current observed result cannot be clearly attributed to the role of immersion and may instead have arisen from a combination of contextual and sensorimotor factors influencing motivational learning. Second, although general PIT has similarities to automatic behavior which could be thought to contribute to the development and formation of habit processes, as the current study did not include a clear outcome devaluation or contingency degradation procedure, we cannot suggest that this observation of increases in general PIT is evidence of habitual control and instead simply reflects outcome-general motivational invigoration which seems to be driven by a number of contributing factors.

As we observed a null finding when examining the interaction between threat group and CF on general and specific PIT effects, this could reflect insufficient sensitivity of the current manipulation to individual differences across the conditions. Therefore, procedural refinement is necessary to better capture the modulation of PIT effects in unique and varying conditions and contexts. Moreover, the interpretation of the physiological stress markers remains limited given the HRV results, making it difficult to reach clear conclusions due to the potential mixed influence of stress and adaptive autonomic flexibility. Similarly, although we observed consistent increases in sAA, it is difficult to disentangle stress elicited solely by the PIT task from contextual and procedural factors, the involvement of VR techniques, and the novelty of the task. Finally, although we aimed to ascertain the translational relevance of PIT procedures across humans and rodents with the comparison of auditory vs. visual cues, our study did not directly measure explicit approach behaviors (e.g., PCA or attentional capture). Implementing these additional elements through an approach behavior as captured by physical movement in reality or in VR could help to further understand cross-species translation. The availability of equipment such as VR-compatible motion platforms and walking disks that allow physical interaction with the digital environments may provide a particularly useful method in this regard (Zhang et al., 2024).

In conclusion, the present findings provide evidence for the persistence of general and specific PIT across diverse and novel task formats, reinforcement contexts, and levels of environmental immersion. Across the various experiments, we found evidence of general PIT being numerically larger in VR procedures compared to the more traditional 2D tasks, while specific PIT remained stable. However, given the non-orthogonal design, the interpretation should remain preliminary rather than constitute direct causal evidence that VR immersion leads to increased general PIT. The current findings are consistent with previous research that suggests that immersive and emotionally-arousing contexts may enhance outcome-general motivational invigoration, while goal-directed responding remains stable (Garofalo and Robbins, 2017; Marzuki et al., 2024; Pool et al., 2015). Future studies should aim to orthogonally manipulate the immersive conditions, reinforcement contexts, and aversive stimuli while additionally including outcome-devaluation procedures to more directly test the relationship between general PIT and habitual control. This could be done by isolating the specific components of the immersive contexts to better understand the mechanisms underpinning the observed effects. Overall, the current study provides evidence of the robustness of PIT across procedures and presents a foundation for the use of immersive paradigms in motivated behavior, while providing evidence of the importance of implementing careful experimental control when utilizing novel and innovative procedures.

## Data Availability

The raw data supporting the conclusions of this article will be made available by the authors, without undue reservation.
