# Supplementary material for: Dawn of the dread: threatening cinematic virtual reality environments enhance general but not specific pavlovian-instrumental transfer
Source: Front Behav Neurosci. 2026 Jun 16;20:1803377. doi: 10.3389/fnbeh.2026.1803377 (PMC13314635; doi:10.3389/fnbeh.2026.1803377)
Supplement: Supplementary file 1 [file Data_Sheet_1.DOCX]

Supplementary Material

# Supplementary Details

Due to the use of food rewards in **Experiment 1 and 2**, participants were excluded if they reported food allergies during prescreening. All participants had normal or corrected-to-normal vision and all participants were between 18-65 years of age without major cardiovascular, neurological, or metabolic illness. Participants were excluded if they were taking any psychoactive or cardiovascular medication, had any known infectious diseases such as HIV, hepatitis, or COVID-19, were pregnant, had specific phobias, or recent drug/alcohol use. Recent caffeine, drug, and alcohol use were recorded in the event of any abnormalities in the alpha-amylase levels in the saliva samples. People with anxiety, depression, and OCD were not excluded, as these are prevalent conditions representative of the general population.

Participants were compensated £8 per person per session in **Experiment 1** and £10 per person per session in **Experiments 2 and 3**. **Experiment 1** received ethical approval from the Faculty Research Ethics Panel at Anglia Ruskin University and the Department of Psychology Ethics Committee at the University of Cambridge (ETH2223-1588; 5161.148) and **Experiment 2 and 3** received ethical approval from both the Department of Psychology Ethics Committee and the Cambridge Psychology Research Ethics Committee (**Experiment 2**: 731.201; **Experiment 3**: PRE.2023.138).

## Pre and Post-Test Questionnaires

In **Experiments 1, 2 and 3** following informed consent, participants completed the demographics questions including self-report trait measures. A post-test questionnaire was administered through Qualtrics, which assessed participants’ knowledge of the contingencies between the cues and the rewards, and the contingencies between responses and the rewards. In addition to these questions in **Experiment 1 and 2**, seven-point Likert scales (from ‘not at all’ to ‘extremely’) of wanting and liking each of the three food rewards were administered alongside a hunger scale before and after the PIT task. These seven-point Likert scales have been demonstrated as a valid and reliable measure of food reward desirability and intake (Rogers & Hardman, 2015). The presentation order of these questions was counterbalanced across participants. In **Experiment 3**, pre and post-test questionnaires also included subjective fear and disgust ratings and level of exposure to video games and horror content.

## Background Sound and Scent Stimuli for Experiment 3

The neutral and ominous soundtracks for Experiment 3 were sourced from open source copy-right free YouTube videos (https://www.youtube.com/watch?v=k3UevKvP9RU and https://www.youtube.com/watch?v=Z6ylGHfLrdI&t=9261s; YouTube, 2025). Moreover, a custom ‘Dungeon Scent’ smell designed by AromaPrime (AromaPrime, 2025) was diffused throughout the room to be delivered throughout the entirety of the general and contamination threat conditions.

## Instructions

The following instructions were displayed on the screen before each of the PIT phases in both the appetitive and aversive designs as detailed below.

### Appetitive Design

#### Instrumental Phase

*‘Someone has said you can get free snacks from our magic box. Use the keyboard to tilt the box to the left [q] or right [p] and learn how to get different snacks! To bank a snack, press the key [b] as soon as you see one! Press [space] to begin.’.*

#### Pavlovian Phase

*‘Our box has been upgraded to make it harder to get free snacks. The magical pictures on the back of the box (or in the auditory phase ‘the magical sounds’) indicate if a snack will fall out. To bank a snack, press the key [b] as soon as you see one! A multiple-choice question will sometimes ask you which snack fell out. Use the keyboard [a b c d] or verbally say the letter (in VR) to indicate the correct answer. Press [space] to begin’.*

#### Transfer Phase

*‘Now you have found one of the special boxes. At random intervals the magical pictures will appear on the back of the box and a snack will fall out. However, this time the snacks are invisible and they will not appear on the screen. Remember what you have learned and get as many snacks as you can. Use the keys to tilt the box left [q] and right [p] and press [space] to begin.’.*

### Aversive Design

#### Instrumental Phase

*‘There has been a zombie outbreak and you have been bitten and infected by a zombie! Press [space] to see the zombie and proceed.’*

*‘If you do nothing, you will turn into a zombie, as you have been infected and the disease is in your blood. You will infect all of your friends and family. Press [space] to see what you will turn into and proceed.’*

*‘Luckily, you have found a pill box that you can shake that contains a cure to get the disease out of your body. Use the keyboard to tilt the box to the left [q] or right [p] and learn how to get the pills! To put a pill in your pocket press the key [b] as soon as you see one! There are different pills and you do not know which ones will save you until the end, so try to get as many as you can! Press [space] to begin.*

#### Pavlovian Phase

*‘This time, there is a new way to get the pills out of the box. The pictures behind the box indicate if a pill will fall out. To put a pill in your pocket press the key [b] as soon as you see one! There are different pills and you do not know which ones will save you until the end, so try to get as many as you can! A multiple-choice question will sometimes ask you which pill fell out. Verbally say one of the following letters [a b c d] to indicate the correct answer. Press [space] to begin.’.*

#### Transfer Phase

*‘You can tilt the box to get pills again. At random intervals the pictures will appear behind the box and you will be able to get the pills. However, this time the pills are invisible and they will not appear on the screen. Remember what you have learned and get as many pills as you can. You will know how many you got and which ones you need to cure yourself at the end. Use the keys to tilt the box left [q] and right [p] and press [space] to begin.’.*

## Photoplethysmogram (PPG) Methods and Analyses

PPG biomarker data was collected for **Experiment 3** using a BioRadio multichannel physiological measurement device (Great Lakes NeuroTechnologies) and the Ninja Theory inbuilt software, namely the ‘Insight Project’. The PPG data was collected using a finger pulse-oxygen monitor placed on the ring finger of the left hand (due to participants still needing to be able to respond with the index finger on the left hand) and the ring and index fingers on the right hand during the task. Participants were requested to sit still as this data was collected. A 5-minute baseline reading was collected, then a reading during the 3-minute threat scenario and a reading for each of the PIT phases (instrumental, pavlovian, and transfer phases).

All preprocessing was conducted in Python, Spyder, Systole package version 6.0.3 (Python, 2025; Legrand & Allen, 2022), and MATLAB version R20204b (MathWorks, 2025). PPG data was collected at a sampling rate of 75 hertz. PPG trace data was segmented using Python for the following phases: baseline, scenario (neutral scenario, contamination scenario or spiders scenario), instrumental phase, pavlovian phase, and transfer phase. Raw PPG data was manually inspected using Python to identify any abnormal data points. As noisy data was identified to clean the signal and minimise any noise and unwanted artefacts, a Butterworth bandpass filter was applied in MATLAB with a passband of 0.5-8 hertz. To determine the Inter-beat (RR) intervals, the signal peaks were captured in the raw data. The RR time series data was interpolated according to the assumptions of the Fourier transformation due to uneven signal sampling. This interpolated RR time series data was then utilised to estimate the power and ratio (Welch’s spectral density estimation) for the low frequency (0.04-0.15 hertz) and high frequency (0.15 – 0.4 hertz) bands.

Heart rate measures (mean HR, median HR, maximum HR, and standard deviation of HR) were extracted from the raw data using Python to provide an overview of the heart rate beats per minute (BPM) data and correct the SDNN. For **Experiment 3** the coefficient of variation SDNN (cvSDNN) was used to examine HRV as an indicator of state-stress. The measure of cvSDNN was chosen over RMSSD (root mean square of successive differences), as this reflects both sympathetic and parasympathetic activity, providing a more comprehensive picture of individual HRV (Shaffer & Ginsberg, 2017). Moreover, the following equation was utilised to determine a minimum and maximum expected heart rate to filter unlikely data points (Tanaka et al., 2001):

$$\boldsymbol{HR}_{\mathbf{(}\boldsymbol{max}\mathbf{)}}\mathbf{= 220 -}\boldsymbol{age}$$

To calculate normalised SDNN or cvSDNN as suggested by Daniel-Watanabe et al. (2025) and de Geus et al. (2019), the following formulae were implemented; first, the inter-beat interval (IBI) in milliseconds (ms) was calculated using the mean heart rate:

$$\text{IBI}\boldsymbol{=}\frac{\boldsymbol{60000}}{\text{Mean HR}}$$

Next, the SDNN was divided by the IBI and multiplied by 100 to yield the cvSDNN value:

$$\boldsymbol{cvSDNN=100}\boldsymbol{\times}\frac{\boldsymbol{SDNN}}{\text{IBI}}$$

The formula below provides an overview of the implemented formula for the normalised SDNN used in the subsequent analyses:

$$\boldsymbol{cvSDNN=100}\boldsymbol{\times}\frac{\boldsymbol{SDNN}}{\boldsymbol{60000}\boldsymbol{/}\text{Mean HR}}$$

Mixed factorial ANOVAs were implemented to examine any main or interaction effects of cvSDNN on the contamination fear group and threat group. Moreover, pairwise comparisons were implemented to examine baseline cvSDNN in comparison to scenario and PIT phase data. Spearman’s rho correlations were additionally implemented to capture any relationships between specific and general PIT, sAA, and self-reported stress and trait scales with the HRV data.

## Salivary Alpha Amylase (sAA) Methods and Analyses

In **Experiment 3**, at the in-person testing session, prior to and following the exposure to the VR scenario and learning task, participants’ saliva samples were taken (twice for each participant) using a passive drool collection technique (duration of 180 seconds per sample) for measurement of alpha-amylase levels (Salimetrics, 2025). Samples were sealed and labelled with assigned participant numbers to protect anonymity in individual tubes. All guidelines according to the Human Tissue Act were adhered to (HTA; license number: 12515) and the experimenters (SB and AY) had full HTA training. Saliva samples were transported to the Biomarker Analysis Laboratory at Anglia Ruskin University (ARU) for processing within the same day of data collection and stored at -20^o^C until analysis.

For **Experiment 3** sAA was collected and corrected for flow rate to report results as **Units of sAA activity/min**. The formula used for this was: **Units of sAA activity/ml x  ml /min (flow rate) = Units of sAA activity/min**. Flow rate was calculated by calculating the volume of saliva produced per sample in millilitres (ml). As the average weight of the sample tube in grams (g) was 2.78g, this weight was divided by the volume (in ml) of the raw samples by number of minutes taken to collect (180 seconds or 3 minutes). The **Units of sAA activity/ml** was then multiplied by the flow rate for each sample resulting in **Units of sAA activity/min**.

Overview of formula for calculating the volume of saliva collected (1g ≈ 1 ml) where V = volume of saliva collected (in ml), W_sample_**​** = weight of the tube with saliva (in g), and W_tube​_ = weight of the empty sample tube = 2.78g:

$$\boldsymbol{V}\mathbf{=}\boldsymbol{W}\mathbf{}_{\boldsymbol{sample}\mathbf{}}\mathbf{-}\boldsymbol{W}_{\boldsymbol{tube}}\mathbf{}\boldsymbol{or} \boldsymbol{V}\mathbf{=}\boldsymbol{W}\mathbf{}_{\boldsymbol{sample}\mathbf{}}\mathbf{-2.78}$$

Overview of formula for calculating flow rate in terms of ml/min where F = sAA flow rate (in ml/min) and t = time taken to collect sample (in min):

$$\boldsymbol{F}=\left( \boldsymbol{W}_{\boldsymbol{sample}}-\mathbf{2}.\mathbf{78} \right)/\boldsymbol{t}$$

Overview of formula for correcting sAA activity for flow rate for sAA units/min:

$$\boldsymbol{sA}\boldsymbol{A}_{\left( \boldsymbol{Units}/\boldsymbol{min} \right)}=\boldsymbol{sA}\boldsymbol{A}_{\left( \boldsymbol{Units}/\boldsymbol{ml} \right)}\times\boldsymbol{F}=\boldsymbol{sA}\boldsymbol{A}_{\left( \boldsymbol{Units}/\boldsymbol{ml} \right)}\times\left( \boldsymbol{W}_{\boldsymbol{sample}}-\mathbf{2}.\mathbf{78} \right)/\boldsymbol{t}$$

Analyses were conducted by grouping sAA data into average **Units of sAA activity/min** per participant at pre-test and average **Units of sAA activity/min** per participant at post-test. 2x2x3 ANOVAs were employed to examine the between-within-groups interactions of pre and post-test, high and low CF, and threat scenario groups. Then a series of paired t-tests were employed to examine the sAA levels as stress indicators at pre and post-test and independent t-tests were employed to examine this across groups. Spearman’s rho correlations were conducted with specific and general PIT, PPG data, and self-reported stress and trait scales.

## Statistical Analyses for PIT

All preprocessing pipelines and statistical analyses were designed and conducted in Python, Spyder version 6.0.3 (Python, 2025).

For all analyses, the significance level was set at p < .05 and was two-tailed. In **Experiment 1**, a 2x2 mixed factorial analysis of variance (ANOVA), with Modality (auditory vs. visual) as a between-subject factor, and Transfer Type (general vs. specific) as a within-subject factor was conducted for the PIT effects and consummatory responding. To determine specific and general PIT, the baseline rate of responding was determined by the total number of key presses outside of cue presentation, divided by the baseline display time, to yield a measure of baseline responding per second. This was deducted from the overall responding per second in the task. For specific PIT, the congruent and incongruent key presses (R_1_ and R_2_ for O_1_ and O_2_ respectively, or R_1_ and R_2_ for O_2_ and O_1_ respectively) were summed with the incongruent (different) deducted from the congruent (same) key presses. For general PIT, responses per second for CS_3_ (rewarded with O_3_) and CS_4_ (rewarded with O_4_ (EMPTY)) were summed, and the responses for CS_4_ were deducted from those for CS_3_. Further ANOVAs were conducted separately for specific PIT (same and different responding by modality) and general PIT (CS_3_ and CS_4_ by modality) to examine the magnitudes of specific cue and general cue responding.

For **Experiment 2**, the same 2x2 ANOVA was employed, however, the between-subjects factor was the appetitive or the aversive PIT context by specific and general PIT including further ANOVAs (specific and general cue responding). **Experiment 3** utilised an advanced version of this analysis, with two between-subjects factors of high or low contamination fear and threat scenario group (spiders, contamination, or neutral) by specific and general PIT. Further ANOVAs were also conducted for this group (specific and general cue responding by contamination fear and scenario groups).

To ensure that contingencies were successfully learned in the instrumental and pavlovian phases for all experiments, participants were required to answer 80% of the contingency questions accurately in the instrumental phase and 75% in the pavlovian phase. This was observed through the answers on the post-test questionnaire. Participants who did not achieve these levels of accuracy during the task were excluded and replaced (**Experiment 1**: *N* = 12; **Experiment 2**: *N* = 6; **Experiment 3**: *N* = 9).

**1.7 Specific and General PIT Effects by Experiment**

**1.7.1 Experiment 1**

A 2×2 mixed factorial ANOVA revealed significant differences in the magnitude of the general and specific PIT across both groups [Type of Transfer: *F*_(1, 58)_ = 6.81, *p* = 0.01, η_p_^2^ = 0.11]. General PIT responding was numerically higher in the visual modality [*M* = 1.49, *SD* = 2.10] compared to the auditory modality [*M* = 0.59, *SD* = 2.91] and specific PIT, responding was higher in the visual modality [*M* = 2.26, *SD* = 1.77] compared to the auditory modality [*M* = 1.44, *SD* = 2.46], but this did not reach statistical significance [Type of Transfer × Modality: *F*_(1, 58)_ = 0.01, *p* = 0.10, η_p_^2^ < .001]. There was also no significant difference in responding according to modality overall [Modality: *F*_(1, 58)_ = 2.73, *p* = 0.10, η_p_^2^ = 0.05] (**Figure 1**).


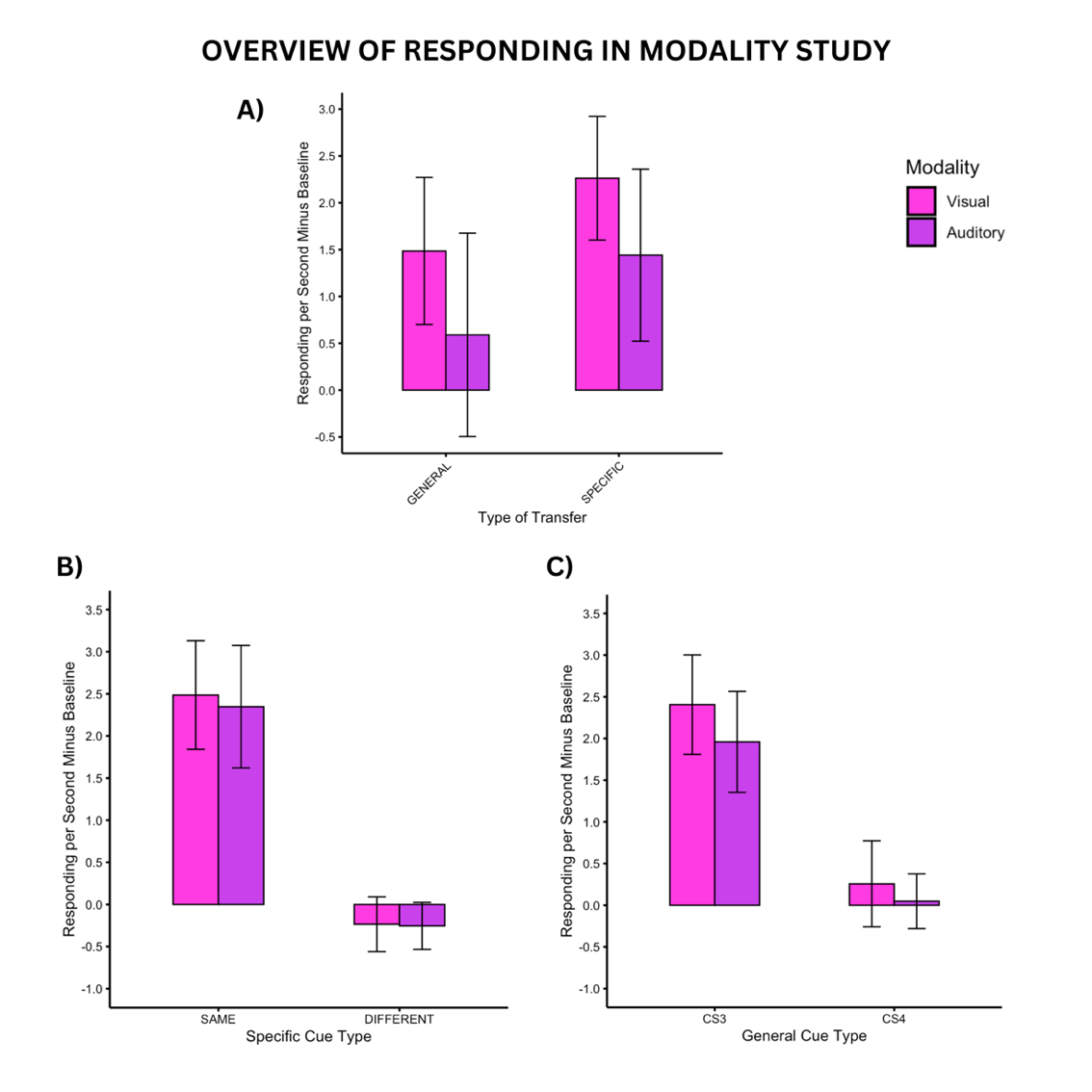


***Figure 1. (A)*** *Overview of overall magnitude of responding per second minus baseline for type of transfer by specific and general PIT according to the visual [Specific, Visual: M = 2.26, SD = 1.77; General, Visual: M = 1.49, SD = 2.10] and auditory modalities [Specific, Auditory: M = 1.44, SD = 2.46; General, Auditory: M = 0.59, SD = 2.9]. The magnitude of the general and specific PIT effects did not differ, either within or between modalities.*

***(B)*** *Overall levels of responding per second minus baseline for specific PIT. Responding was greater on the key that shared an outcome with the cue presented (‘same’) as compared to the key that shared an outcome with a different cue (‘different’). The effect was the same, regardless of whether the cues were visual [Same, Visual: M = 2.47, SD = 1.72; Different, Visual: M = -0.24, SD = 0.87] or auditory [Same, Auditory: M = 2.35, SD = 1.95; Different, Auditory: M = -0.25, SD = 0.75].*

***(C)*** *Overall levels of responding per second minus baseline for general PIT. Responding was greater when CS_3_, which had been associated with a reward during pavlovian training, was presented compared to CS_4_, which had been associated with an ‘EMPTY’ box during pavlovian training. The effect here was also the same, regardless of whether the cues were visual [CS_3_, Visual: M = 2.41, SD = 1.60; CS_4_, Visual: M = 0.26, SD = 1.38] or auditory [CS_3_, Auditory: M = 1.96, SD = 1.62; CS_4_, Auditory: M = 0.05, SD = 0.88]. Y-axis is Responding per Second Minus Baseline. Data are represented as mean ± 95% confidence intervals.*

### Specific Transfer

To investigate the difference in responding to congruent and incongruent cues, a 2x2 mixed factorial ANOVA was used to compare specific cue responding (same vs. different) and modality (visual vs. auditory) (**Figure 1B**). Participants responded more on the key congruent with the previous outcome [Same, Auditory: *M* = 2.35, *SD* = 1.95; Same, Visual: *M* = 2.47, *SD* = 1.72] as compared to the key that was incongruent [Different, Auditory: *M* = -0.25, *SD* = 0.75; Same, Visual: *M* = -0.24, *SD* = 0.87], [Specific Cue Responding: *F*_(1, 58)_ = 124, *p* < .001, η_p_^2^ = 0.68]. This did not differ between the visual and auditory conditions [Modality: *F*_(1, 58)_ = 0.08, *p* = 0.78, η_p_^2^ = 0.001; Specific Cue Responding x Modality: *F*_(1, 58)_ = 0.06, *p* = 0.81, η_p_^2^ = 0.001], indicating that specific transfer does not differ according to modality.

### General Transfer

General PIT (**Figure 1C**) was assessed by comparing responding during the presentation of CS_3_ (which had been associated with a reward not presented during the instrumental phase) to responding during the presentation of CS_4_ (which had been associated with the ‘EMPTY’ outcome during the instrumental phase). A 2×2 mixed factorial ANOVA showed that responding was greater for CS_3_ [Auditory: *M* = 1.96, *SD* = 1.62; Visual: *M* = 2.41, *SD* = 1.60] than CS_4_ [Auditory: *M* = 0.05, *SD* = 0.88; Visual: *M* = 0.26, *SD* = 1.38], [General Cue Responding: *F*_(1, 58)_ = 89.80, *p* < .001, η_p_^2^ = 0.61], regardless of whether the cues were visual or auditory [Modality: *F*_1, 58)_ = 1.26, *p* = 0.27, η_p_^2^ = 0.02; General Cue Responding x Modality: *F*_(1, 58)_ = 1.26, *p* = 0.27, η_p_^2^ = 0.005].

**1.7.2 Experiment 2**

A 2x2 mixed measures ANOVA compared specific and general PIT values between the appetitive and aversive conditions (**Figure 2A**). There were no differences in general and specific PIT effects [Transfer Type: *F*_(1, 38)_ = 0.29, *p* = 0.59, η_p_^2^ = 0.008] which persisted across both appetitive [General, Appetitive: *M* = 2.46, *SD* = 1.37; Specific, Appetitive: *M* = 2.21, *SD* = 1.52] and aversive conditions [General, Aversive: *M* = 1.86, *SD* = 1.71; Specific, Aversive: *M* = 1.76, *SD* = 2.41] [Context: *F*_(1, 38)_ = 1.29, *p* = 0.26, η_p_^2^ = 0.33; Context x Transfer Type: *F*_(1, 38)_ = 0.06, *p* = 0.81, η_p_^2^ = 0.002].

**
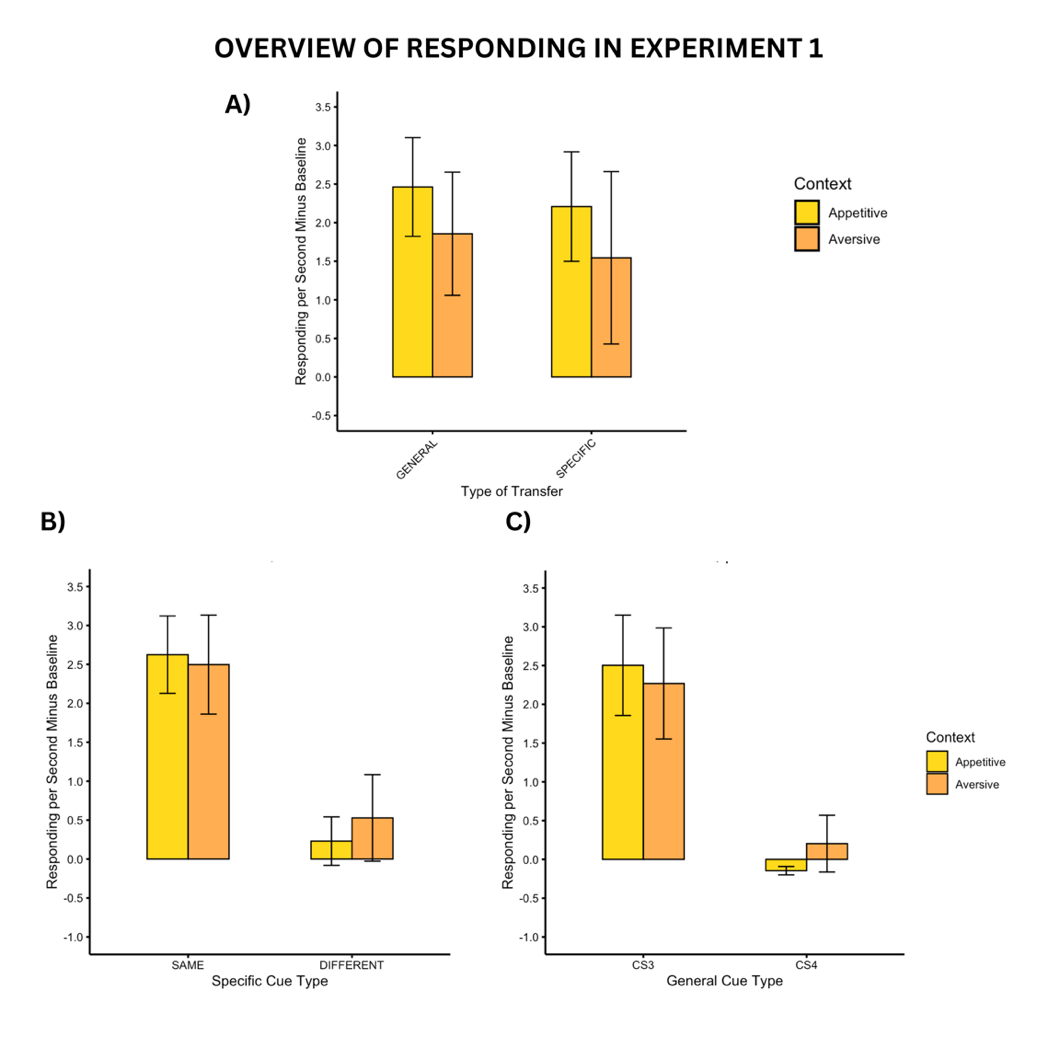
**

***Figure 2. (A)*** *Overview of overall magnitude of responding per second minus baseline for type of transfer by specific and general PIT by appetitive [General, Appetitive: M = 2.46, SD = 1.37; Specific, Appetitive: M = 2.21, SD = 1.52] or aversive context [General, Aversive: M = 1.86, SD = 1.71; Specific, Aversive: M = 1.76, SD = 2.41]. There were no differences in the magnitude of general and specific PIT effects by context.*

***(B)*** *Overall levels of responding per second minus baseline for specific PIT. Specific transfer effects are depicted through the interaction between specific cue responding (responding per second for ‘same’ and ‘different’ responses minus baseline) and context (appetitive and aversive). There was no difference in responding for appetitive [Same, Appetitive: M = 2.63, SD = 1.06; Different, Appetitive: M = 0.23, SD = 0.67] or aversive contexts [Same, Aversive: M = 2.50, SD = 1.36; Different, Aversive: M = 0.53, SD = 1.19], however, there was a significant increase in ‘same’ responses over ‘different’ ones.*

***(C)*** *Overall levels of responding per second minus baseline for general PIT. General transfer effects are depicted through the interaction between general cue responding (responding per second for CS_3_ and CS4) and Context. There was no difference in responding for appetitive [CS_3_, Appetitive: M = 2.50, SD = 1.38; CS_4_, Appetitive: M = -0.15, SD = 0.12] or aversive contexts [CS_3_, Aversive: M = 2.27, SD = 1.53; CS_4_, Aversive: M = 0.20, SD = 0.78], however, there was a significant increase in CS_3_ responses over responses for CS_4_. Y-axis is Responding per Second Minus Baseline. Data are represented as mean ± 95% confidence intervals.*

### Specific Transfer

To determine whether specific transfer differed between the two conditions, a 2x2 mixed measures ANOVA was conducted with specific cue responding (responding per second for the same or different reward) and context (appetitive and aversive) (**Figure 2B**). Participants responded congruently on the buttons that were previously reinforced with rewards (‘same’) from the instrumental phase compared to those that were not (‘different’) [Specific Cue Responding: *F*_(1, 38)_ = 45.94, *p <* .001, η_p_^2^ = 0.55] and this persisted regardless of appetitive [Same, Appetitive: *M* = 2.63, *SD* = 1.06; Different, Appetitive: *M* = 0.23, *SD* = 0.67] or aversive condition [Same, Aversive: *M* = 2.50, *SD* = 1.36; Different, Aversive: *M* = 0.53, *SD* = 1.19] [Context: *F*_(1, 38)_ = 0.43, *p* = 0.52, η_p_^2^ = 0.01; Specific Cue Responding x Context: *F*_(1, 38)_ = 0.44, *p* = 0.51, η_p_^2^ = 0.01]. These results present evidence of a significant difference between same and different cue responding demonstrating specific PIT, which was similar in both the appetitive and aversive contexts.

### General Transfer

To determine whether general transfer differed between the two conditions, a 2x2 mixed measures ANOVA was conducted with General Cue Responding (responding per second for CS_3_ or CS_4_) and Context (appetitive and aversive) (**Figure 2C**). Responding was much greater for cues that were reinforced with a rewarding outcome in the pavlovian phase over those that were not [General Cue Responding: *F*_(1, 38)_ = 85.89, *p <* .001, η_p_^2^ = 0.69] regardless of the appetitive [CS_3_, Appetitive: *M* = 2.50, *SD* = 1.38; CS_4_, Appetitive: *M* = -0.15, *SD* = 0.12] or aversive condition [CS_3_, Aversive: *M* = 2.27, *SD* = 1.53; CS_4_, Aversive: *M* = 0.20, *SD* = 0.78] [Context: *F*_(1, 38)_  = 0.06, *p* = 0.81, η_p_^2^ = 0.002; General Cue Responding x Context: *F*_(1, 38)_ = 1.31, *p* = 0.26, η_p_^2^ = 0.03]. These results present evidence of a significant difference between responding for CS_3_ and CS_4_ providing evidence for general PIT, however no significant difference between the two context conditions.

**1.7.3 Experiment 3**

A 2x2x3 mixed measures ANOVA compared specific and general PIT values between the three threat conditions (spiders, contamination and neutral; **Figure 3A**) and the low and high CF groups (**Figure 4A**). There was only a main effect of transfer type which indicates differences in general and specific PIT effects [Transfer Type: *F*_(1, 90)_ = 12.77, *p* < .001, η_p_^2^ = 0.12]. There were no other significant main effects or interactions which persisted across all conditions [CF: *F*_(1, 90)_ = 0.54, *p* = 0.47, η_p_^2^ = 0.006; Threat Group: *F*_(2, 90)_ = 1.01, *p* = 0.37, η_p_^2^ = 0.02; CF x Threat Group: *F*_(2, 90)_ = 0.65, *p* = 0.53, η_p_^2^ = 0.01; Transfer Type x CF: *F*_(2, 90)_ = 0.18, *p* = 0.67, η_p_^2^ = 0.002; Transfer Type x Threat Group: *F*_(2, 90)_ = 1.18, *p* = 0.311, η_p_^2^ = 0.03; Transfer Type x CF x Threat Group: *F*_(2, 90)_ = 0.06, *p* = 0.94, η_p_^2^ = 0.001].

Moreover, the overall magnitudes of responding for both general and specific PIT (by group: **Figure 3A**; by CF: **Figure 4A**) and for same over different responses (specific PIT by group: **Figure 3B**; by CF: **Figure 4B**) and CS_3_ over CS_4_ minus baseline responding (ITI) (general PIT by group: **Figure 3C**; by CF: **Figure 4C**) are illustrated below. The expected trends were once again observed with higher responding for same compared to different responses and CS_3_ over CS_4_ resulting in strong specific and general PIT effects) (**Figure 3 and Figure 4**).


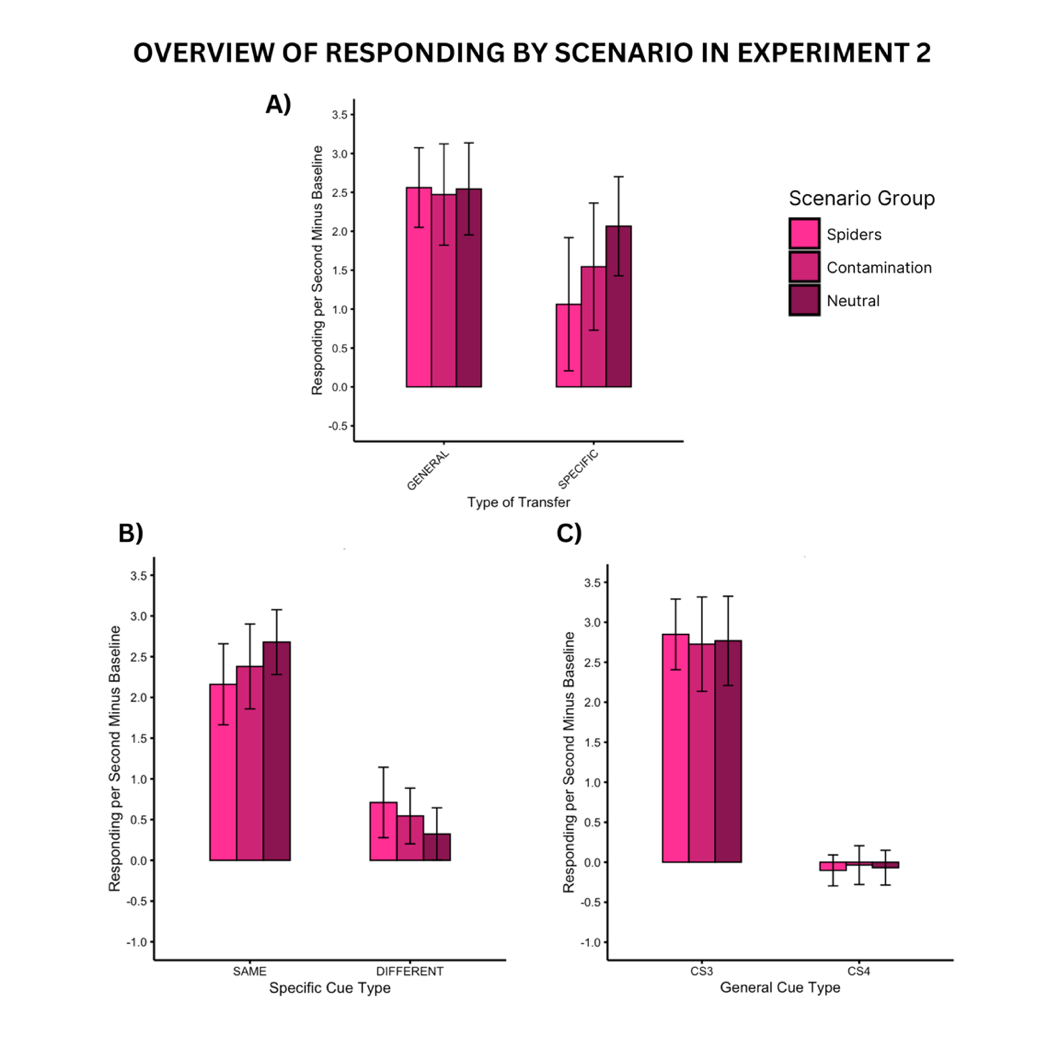


***Figure 3 (A)*** *Overview of the overall magnitude of responding for general and specific PIT by threat scenario group (Spiders [Spiders, General: M = 2.56, SD = 1.42; Spiders, Specific: M = 1.06, SD = 2.38], Contamination [Contamination, General: M = 2.47, SD = 1.80; Contamination, Specific: M = 1.55, SD = 2.27] and Neutral [Neutral, General: M = 2.55, SD = 1.64; Neutral, Specific: M = 2.07, SD = 1.77]) with no significant between groups differences.*

***(B)*** *The overall magnitude of responding for specific PIT was consistent across groups: when responding for cues that were ‘same’ or ‘different’ to the rewarded ones by threat group (Spiders [Spiders, Same: M = 2.16, SD = 1.38; Spiders, Different: M = 0.71, SD = 1.20], Contamination [Contamination, Same: M = 2.38, SD = 1.45; Contamination, Different: M = 0.55, SD = 0.95] and Neutral [Neutral, Same: M = 2.68, SD = 1.10; Neutral, Different: M = 0.32, SD = 0.89]) with no significant between-groups differences.*

***(C)*** *The overall magnitude of responding for general PIT was the same across groups: when cues were CS_3_ or CS_4_ by threat group Spiders [Spiders, CS_3_: M = 2.85, SD = 1.23; Spiders, CS_4_: M = -0.10, SD = 0.54], Contamination [Contamination, CS_3_: M = 2.73, SD = 1.64; Contamination, CS_4_: M = -0.04, SD = 0.67] and Neutral [Neutral, CS_3_: M = 2.77, SD = 1.55; Neutral, CS_4_: M = -0.07, SD = 0.60]). Here, between-groups differences were also not significant. The Y-axis is Responding per Second Minus Baseline. Data are represented as mean ± 95% confidence intervals.*


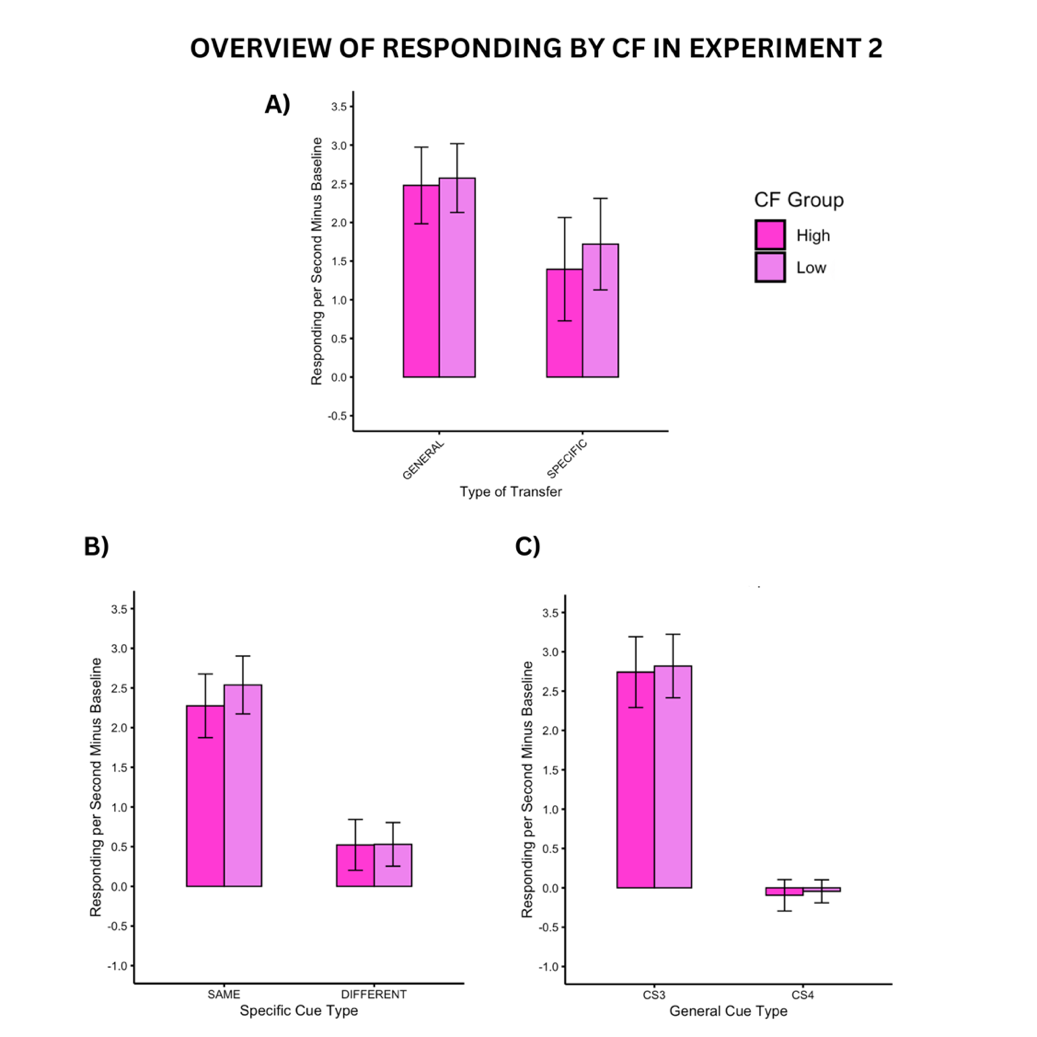


***Figure 4 (A)*** *Overview of the overall magnitude of responding for general and specific PIT by high [High CF, General: M = 2.48, SD = 1.70; High CF, Specific: M = 1.40, SD = 2.30] and low CF [Low CF, General: M = 2.57, SD = 1.53; Low CF, Specific: M = 1.72, SD = 2.04] did not differ between-groups.*

***(B)*** *The overall magnitude of responding for specific PIT did not differ between groups either: when responding for cues that were ‘same’ or ‘different’ to the rewarded ones by high [High CF, Same: M = 2.28, SD = 1.38; High CF, Different: M = 0.52, SD = 1.10] and low CF [Low CF, Same: M = 2.54, SD = 1.26; Low CF, Different: M = 0.53, SD = 0.95] with no difference between-groups.*

***(C)*** *The overall magnitude of responding for general PIT was the same between groups: when cues were CS_3_ or CS_4_ by CF group with no significant between-groups differences between the high [High CF, CS_3_: M = 2.74, SD = 1.55; High CF, CS_4_: M = -0.09, SD = 0.69] and low [Low CF, CS_3_: M = 2.82, SD = 1.39; Low CF, CS_4_: M = -0.04, SD = 0.50] groups. The Y-axis is Responding per Second Minus Baseline. Data are represented as mean ± 95% confidence intervals.*

Specific Transfer

To determine whether specific transfer differed between high and low CF and the threat groups (spiders, contamination and neutral), a 2x2x3 mixed measures ANOVA was conducted with specific cue responding (responding per second for the same or different reward), threat group (spiders, contamination and neutral) (**Figure 4B**), and CF low and high (**Figure 5B**).

Participants once again responded congruently on responses that were reinforced with rewards from the instrumental phase to those that were not [Specific Cue Responding: *F*_(1, 90)_ = 72.68, *p <* .001, η_p_^2^ = 0.45] and this persisted regardless of condition with no between-groups interactions [CF: *F*_(1, 90)_  = 1.72, *p* = 0.19, η_p_^2^ = 0.02; Threat Group: *F*_(2, 90)_  = 0.14, *p* = 0.87, η_p_^2^ = 0.003; CF x Threat Group: *F*_(1, 90)_ = 0.33, *p* = 0.72, η_p_^2^ = 0.007; Specific Cue Responding x CF: *F*_(1, 90)_ = 0.34, *p* = 0.56, η_p_^2^ = 0.004; Specific Cue Responding x Threat Group: *F*_(1, 90)_ = 1.41, *p* = 0.25, η_p_^2^ = 0.03; Specific Cue Responding x CF x Threat Group: *F*_(1, 90)_ = 0.19, *p* = 0.83, η_p_^2^ = 0.004]. These results present evidence of a significant difference between same and different cue responding, which was similar in both the high and low CF groups and across all three threat conditions (spiders, contamination and neutral).

### General Transfer

To determine whether general transfer differed between high and low CF and the threat groups (spiders, contamination, and neutral), a 2x2x3 mixed measures ANOVA was conducted with general cue responding (responding per second for the same or different reward), threat group (spiders, contamination and neutral) (**Figure 3C**), and CF low and high (**Figure 4C**).

Participants once again responded congruently on responses that were reinforced with rewards from the instrumental phase to those that were not [General Cue Responding: *F*_(1, 90)_ = 279.18, *p <* .001, η_p_^2^ = 0.76] and this persisted regardless of condition with no between-groups interactions [CF: *F*_(1, 90)_  = 0.17, *p* = 0.69, η_p_^2^ = 0.002; Threat Group: *F*_(2, 90)_ = 0.01, *p* = 0.99, η_p_^2^ < .001; CF x Threat Group: *F*_(1, 90)_ = 0.86, *p* = 0.43, η_p_^2^ = 0.02; General Cue Responding x CF: *F*_(1, 90)_ = 0.01, *p* = 0.94, η_p_^2^ < .001; General Cue Responding x Threat Group: *F*_(1, 90)_ = 0.10, *p* = 0.90, η_p_^2^ = 0.002; Specific Cue Responding x CF x Threat Group: *F*_(1, 90)_ = 0.52, *p* = 0.60, η_p_^2^ = 0.01]. These results present evidence of a significant difference between CS_3_ and CS_4_ cue responding, which was similar in both the high and low CF groups and across all three threat conditions (spiders, contamination and neutral).

**1.8 Safety Protocol**

In case of any adverse reactions to the tasks in any of the three experiments, there was a strict safety protocol in place where the task would be immediately terminated, and the participant would come out of the headset and would be told to sit still until dizziness subsided in the case of cybersickness. In case of any medical emergency or need of medical care, the university medics onsite at the Department of Psychology at the University of Cambridge would be called to the testing room and care would be administered.
